# Factors associated with reversals of COVID-19 vaccination willingness: Results from two longitudinal, national surveys in Japan 2021-2022

**DOI:** 10.1016/j.lanwpc.2022.100540

**Published:** 2022-07-21

**Authors:** Cyrus Ghaznavi, Daisuke Yoneoka, Takayuki Kawashima, Akifumi Eguchi, Michio Murakami, Stuart Gilmour, Satoshi Kaneko, Hiroyuki Kunishima, Wataru Naito, Haruka Sakamoto, Keiko Maruyama-Sakurai, Arata Takahashi, Yoshihiro Takayama, Yuta Tanoue, Yoshiko Yamamoto, Tetsuo Yasutaka, Hiroaki Miyata, Shuhei Nomura

**Affiliations:** a Department of Health Policy and Management, School of Medicine, Keio University, Tokyo, Japan; b Medical Education Program, Washington University School of Medicine in St Louis, Saint Louis, USA; c Department of Global Health Policy, Graduate School of Medicine, The University of Tokyo, Tokyo, Japan; d Tokyo Foundation for Policy Research, Tokyo, Japan; e Infectious Disease Surveillance Center, National Institute of Infectious Diseases, Tokyo, Japan; f Department of Mathematical and Computing Science, Tokyo Institute of Technology, Tokyo, Japan; g Center for Preventive Medical Sciences, Chiba University, Chiba, Japan; h Center for Infectious Disease Education and Research, Osaka University, Osaka, Japan; i Graduate School of Public Health, St. Luke's International University, Tokyo, Japan; j Department of Ecoepidemiology, Institute of Tropical Medicine, Nagasaki University, Nagasaki, Japan; k Department of Infectious Diseases, St. Marianna University School of Medicine, Kanagawa, Japan; l Research Institute of Science for Safety and Sustainability, National Institute of Advanced Industrial Science and Technology (AIST), Tsukuba, Ibaraki, Japan; m Department of Hygiene and Public Health, Tokyo Women's Medical University, Tokyo, Japan; n Department of Healthcare Quality Assessment, Graduate School of Medicine, The University of Tokyo, Tokyo, Japan; o Department of International Health and Medical Anthropology, Institute of Tropical Medicine, Nagasaki University, Nagasaki, Japan; p Division of Infectious Diseases, Okinawa Prefectural Chubu Hospital, Uruma, Okinawa, Japan; q Institute for Business and Finance, Waseda University, Tokyo, Japan; r National Center for Child Health and Development, Tokyo, Japan; s Research Institute for Geo-Resources and Environment, National Institute of Advanced Industrial Science and Technology (AIST), Ibaraki, Japan

**Keywords:** Japan, COVID-19, Vaccine, Vaccination, Vaccine hesitancy

## Abstract

**Background:**

Research characterizing changes of heart with respect to vaccine intention is scarce, and very little research considers those who were initially vaccine willing but became hesitant. Here, we sought to assess the factors related to reversals of vaccine willingness.

**Methods:**

We conducted a longitudinal, national survey on vaccination intention among Japanese adults aged 20 years or older, with the first questionnaire performed in February-March 2021 (*N* = 30,053) and the follow-up in February 2022 (*N* = 19,195, response rate 63.9%). The study population comprised those who reported vaccine willingness in the first survey, with the outcome variable being development of vaccine hesitancy at follow-up. We performed a regression analysis of vaccination status using sociodemographic, health-related, psychologic/attitudinal, and information-related variables as predictors. We used the sparse group minimax concave penalty (MCP) to select the optimum group of covariates for the logistic regression.

**Findings:**

Of 11,118 (57.9%) respondents who previously expressed interest in vaccination, 10,684 (96.1%) and 434 (3.9%) were in the vaccine willing and hesitant groups, respectively. Several covariates were found to significantly predict vaccine hesitancy, including marital status, influenza vaccine history, COVID-19 infection/testing history, engagement in COVID-19 preventive measures, perceived risks/benefits of the COVID-19 vaccine, and attitudes regarding vaccination policies and norms. The use of certain information sources was also associated with vaccine hesitancy.

**Interpretation:**

Sociodemographic, health-related, psychologic/attitudinal, and information-related variables predicted the development of vaccine hesitancy among those with prior willingness. Most of these predictors were also associated with vaccination status.

**Funding:**

The present work was supported in part by a grant from the Kanagawa Prefectural Government of Japan and by AIST government subsidies.

**Research in context**

**Evidence before this study**

We searched PubMed Central for articles published in English from 1 January 2020 to 7 April 2022 with the following keywords: (“COVID-19” OR “SARS-CoV-2”) AND “vaccin*” AND (“intent*” or “hesitan*” or “willing*”) AND (“follow-up” or “longitudinal”). Our search yielded 147 articles. Few studies examined reversals in COVID-19 vaccine intentions, and of those, the large majority were limited to specific subpopulations (e.g., healthcare workers, those living with multiple sclerosis, etc.). One national US study assessed vaccination outcomes using longitudinal surveys in April/May and June/July 2021 and found that 17.0% of those initially desiring vaccination became unsure or willing while 14.5% of those initially unsure or unwilling became willing or already received vaccination. Another US study found that among those initially willing to receive vaccination in October 2020, between 6-19% (depending on race/ethnicity) had not yet been inoculated by July 2021. Of the available evidence, most focus on the shift from being unsure/unwilling to willing, and very few consider individuals who were initially willing but ultimately forewent vaccination. Thus, definitive characterizations of these populations who experienced changes of heart remain elusive.

**Added value of this study**

We present the findings of a one-year, longitudinal follow-up survey of COVID-19 vaccine intention among 30,053 Japanese adults aged 20 years of older that yielded 19,195 responses regarding vaccination status. 11,118 (57.9%) respondents previously expressed interest in vaccination: of those, 10,684 (96.1%) had been vaccinated (or made an appointment for vaccination) or remained vaccine willing and 434 (3.9%) developed vaccine hesitancy at one-year follow-up. We used logistic regression with a sparse group minimax concave penalty to determine which factors were associated with vaccine hesitancy despite prior willingness. Several sociodemographic, health-related, psychologic/attitudinal, and information-related variables covariates were found to significantly predict vaccine hesitancy among previously willing respondents one-year after Japan's vaccine rollout.

**Implications of all the available evidence**

Vaccine hesitancy remains a major barrier to achieving herd immunity against COVID-19 globally, especially in the wake of new variants and easing of activity restrictions. Those who previously expressed the desire to receive the COVID-19 vaccine but ultimately became hesitant constitute an overlooked subpopulation in the vaccine hesitancy literature and in vaccination campaigns, which tend to emphasize those who were initially unsure or unwilling. Our findings constitute one of the first characterizations of those who previously demonstrated vaccine willingness but became hesitant using a large, national sample with adequate follow-up. This subpopulation merits targeted attention in future public health campaigns.

Alt-text: Unlabelled box

## Introduction

The rapid development of effective vaccinations has become one of the longstanding legacies of the COVID-19 pandemic. As of April 7, 2022, 36 COVID-19 vaccines have been approved for use globally,^1^ and approximately 11·34 billion doses have been administered worldwide.^2^ The vaccine rollout in Japan began slowly compared to other high-income nations but eventually picked up speed in large part due to the hosting of the Tokyo 2020 Summer Olympic Games.^3^ As of the same date, three vaccine formulations have received governmental approval (Moderna, Pfizer, and AstraZeneca), and 75·0% of the population has received a two-dose vaccination series.^4^ The approval and distribution of booster vaccinations took considerably longer in Japan than other similar nations: 43·5% of Japanese residents have received their third dose, which can be administered at least six months after receiving your second inoculation in Japan. Considering the effects of incomplete immunity in the setting of new variants, discussions regarding the use of a second booster inoculation (i.e., fourth dose) are already underway.^5^

Globally, vaccine hesitancy has proven to be a major obstacle in preventing the widespread administration of vaccines and the achievement of herd immunity. Multi-country surveys have shown wide country-to-country variation in vaccine acceptance (31-89%).^6^^,^^7^ Systematic reviews of vaccine intention have found that various socioeconomic variables such as gender, age, race/ethnicity, education, income, and place of residence are associated with COVID-19 vaccine hesitancy.^8^^,^^9^ Prior influenza vaccine history, trust in governmental institutions, compliance with subjective norms, and other behaviors and psychologic dispositions have also been shown to be associated with vaccine intention.^7^^,^^10^^,^^11^ Concerningly, the rate of vaccine hesitancy seems to have increased as the pandemic has progressed.^9^

A multi-country analysis of vaccine confidence in 149 countries ranked Japan as among the countries with the lowest vaccine confidence in the world.^12^ The vaccine hesitancy movement in Japan gained momentum in 2013 when the Ministry of Health, Labour and Welfare withdrew its recommendation for the human papillomavirus (HPV) vaccine after mass media reporting of unconfirmed adverse effects in young girls post-inoculation; HPV vaccination rates plummeted from approximately 70% to less than 1% soon after.^13^ Previous research on COVID-19 vaccine hesitancy in Japan has found that prior to the vaccine rollout, 32·9% and 11·0% of Japanese adults were unsure or unwilling to vaccinate, respectively.^14^ Factors associated with vaccine hesitancy in Japan include being female, young age, low socioeconomic status, low trust in scientists and public institutions, and the use of certain information sources such as YouTube.^14^^,^^15^ These data have been crucial for formulating appropriate public health messaging for vaccination campaigns.

Notably, there has been a dearth of data regarding the characteristics of individuals who have reversals of vaccine intention even though this population constitutes a prime target for public health messaging. In addition to a handful of other studies assessing limited population such as health care workers,^16^, ^17^, ^18^ the elderly,^19^ refugees,^20^ COVID-19 recovered patients,^21^ and people living with multiple sclerosis,^22^^,^^23^ only two studies have attempted to assess vaccine changes of heart using actual vaccination as the primary outcome. Szilagyi et al. (2021) assessed vaccination outcomes using longitudinal surveys in April/May and June/July 2021 and found that 17·0% of those initially desiring vaccination became unsure or unwilling while 14·5% of those initially unsure or unwilling became willing or already received vaccination.^24^ A similar study by Rane et al. (2021) found that among those initially willing to receive vaccination in October 2020, between 6-19% (depending on race/ethnicity) had not yet been inoculated by July 2021.^25^ Notably, the overwhelming majority of these studies focus on those who went from being unsure/unwilling to willing/vaccinated, but very few consider individuals who converted from willing to unsure/unwilling. Moreover, as most evidence is based on cross-sectional surveys, short follow-up periods, and specific sub-populations, definitive characterizations of these change-of-heart populations remain elusive.

In this study, we seek to identify factors associated with the development of vaccine hesitancy despite initial willingness by using a large, national sample of Japanese adults with a one-year follow-up period. We use longitudinal survey data from February 2021, prior to the vaccine rollout in Japan, and February 2022, after the start of booster vaccination campaigns, to assess the characteristics of those who initially described willingness to obtain the vaccine but had become hesitant one year later.

## Methods

### Survey respondents

Details concerning the study participants recruited via Cross Marketing Inc., an online survey company, are reported elsewhere.^14^ Briefly, the first of two longitudinal surveys was conducted between 26 February and 4 March 2021, before COVID-19 vaccination officially began in Japan. The survey employed a quota sampling method based on age, gender, and prefecture of residence such that the sample reflected the 2015 National Census of Japan with respect to these characteristics. The final sample size was 30,053, comprised of adults aged 20 and older nationwide. The second survey was made available to the respondents of the first survey and was conducted approximately one year later, between 4 and 24 February 2022, after third-dose vaccinations had become available in Japan. The second survey employed the same quota sampling method used in the first survey, assuming a 70% response rate: ultimately, 19,195 responses were collected (response rate 63·9%). For both surveys, respondents were required to answer all questions such that no missing responses were generated.

### Study population

In the first survey, respondents were asked whether they intend to receive the COVID-19 vaccination once made available (‘yes,’ ‘no,’ or ‘unsure’). In the second survey, respondents were asked whether they have already received (or made an appointment to receive) at least one dose of the vaccine (‘yes,’ ‘interested in vaccination but have yet to make an appointment,’ ‘unsure regarding vaccination,’ or ‘do not intend to be vaccinated’). Those who were already vaccinated or have made an appointment selected the same answer choice (‘yes’). The current study assessed those who initially answered ‘yes’ on the first survey. For simplicity, those who answered ‘yes’ or ‘interested in vaccination but have yet to make an appointment’ on the second survey are hereafter referred to as ‘vaccine willing,’ while the remainder are hereafter referred to as ‘vaccine hesitant.’

### Survey design

Details concerning the specific questions asked in the first survey, and the means by which they were developed are explained in detail elsewhere.^14^ Briefly, both the first and second surveys are comprised of three parts: (1) health-related questions; (2) psychological attributes and vaccine attitudes; and (3) the use of and trust in COVID-19 information sources. The first survey also covered socioeconomic characteristics, which were not included in the longitudinal follow-up (with the exception of prefecture of residence), assuming no significant changes during the interim period. All survey items were closed-ended and consisted of single- or multiple-response questions.

### Descriptive analysis

For the present study, we considered socioeconomic characteristics (except prefecture of residence), underlying health conditions of the respondent or other household members, and past influenza/routine vaccine behavior from the first survey. From the second survey, we considered all variables queried with the exception of those that did not apply to those who initially expressed vaccine willingness (e.g., “For those of you who were initially unsure/unwilling, what made you change your mind and decide to get vaccinated?”) or those that were redundant with prior questions (listed in Supplementary Material). We calculated proportions or means with standard deviations for all variables, stratified by vaccination status at one year. Chi-squared tests (for proportions) and Student's t-tests (for means) were used to test for statistically significant differences between the two groups. Where possible, to decrease variable burden for the subsequent regression analysis, categorical variables were reclassified into fewer categories: for example, prefecture of residence (47 prefectures) was reclassified to regions (6 regions). A full list of reclassifications is available in the Supplementary Material. By the same reasoning, Likert scale-type questions relating to trust and use of information sources were treated as continuous variables; all other Likert scale-type questions (i.e., health-related or psychological attributes and vaccine attitudes) were treated as categorical variables. For simplicity, the descriptive analysis presented in the main text is limited to sociodemographic variables; the corresponding table for all remaining variables can be found in the Supplementary Material.

### Regression analysis

The outcome variable of the current study was whether one remained vaccine willing (coded as 0) or developed vaccine hesitancy (coded as 1) as of the second survey among those who previously endorsed vaccine willingness. Because our dataset includes many possible covariates and including all of them in a predictive model would certainly result in overfitting and collinearity among covariates, we employed a variable selection step to determine which covariates significantly and effectively predicted remaining unvaccinated despite prior vaccine willingness. In this study, we utilized the sparse group minimax concave penalty (MCP) to select the optimum group of covariates that best predict the outcome variable while encouraging sparsity in the final number of covariates and discouraging multicollinearity. The MCP penalty has been shown to outperform other penalties, such as the group LASSO (least absolute shrinkage and selection operator).^26^ The questionnaire data has a group structure for all categorical covariates: for example, the question ‘Do you receive influenza vaccinations’ included the responses ‘every year,’ ‘every few years,’ and ‘rarely or never.’ These options are important relative to one another and are not independent; thus a group selection method that assesses categorical covariates with groups of variables as opposed to individual, independent variables is necessary. The group MCP can identify important groups while maintaining the interpretability of regression results; continuous variables are handled in the standard fashion. Our data includes 168 groups (i.e., questions or covariates) that consist of 216 variables (i.e., the sum total of [the number of answer choices for each categorical variable – 1] + the number of continuous variables). Once the optimal penalty was estimated, the final regression coefficients were estimated without the penalty using logistic regression to debias the shrunken estimates in the coefficients due to the penalty.^27^ All analyses were performed in *R* version 4.1.1 (*grpreg* package)^28^ and Stata version 17.0. The results of the final logistic regression using only the selected covariates are presented here.

### Sensitivity analysis

To test whether changing the primary outcome of this study from vaccination intention to vaccination status had any substantial effect on the results of the regression analyses, we performed a sensitivity analysis using vaccination status as the outcome variable. In the second survey, those who selected ‘already received (or made an appointment to receive) at least one dose of the vaccine’ were classified as ‘vaccinated’ (coded as 0); those who selected ‘interested in vaccination but have yet to make an appointment,’ ‘unsure regarding vaccination,’ or ‘do not intend to be vaccinated’ were classified as ‘unvaccinated’ (coded as 1). The logistic regression with sparse group minimax concave penalty was then repeated using this new classification system.

### Data sharing

The datasets generated during and/or analyzed during the current study are not publicly available due to ethical considerations but are available from the corresponding author on reasonable request.

### Role of the funding source

The funder of the study had no role in study design, data collection, data analysis, data interpretation, or the writing of this report.

## Results

### Descriptive analysis

**Figure 1:**
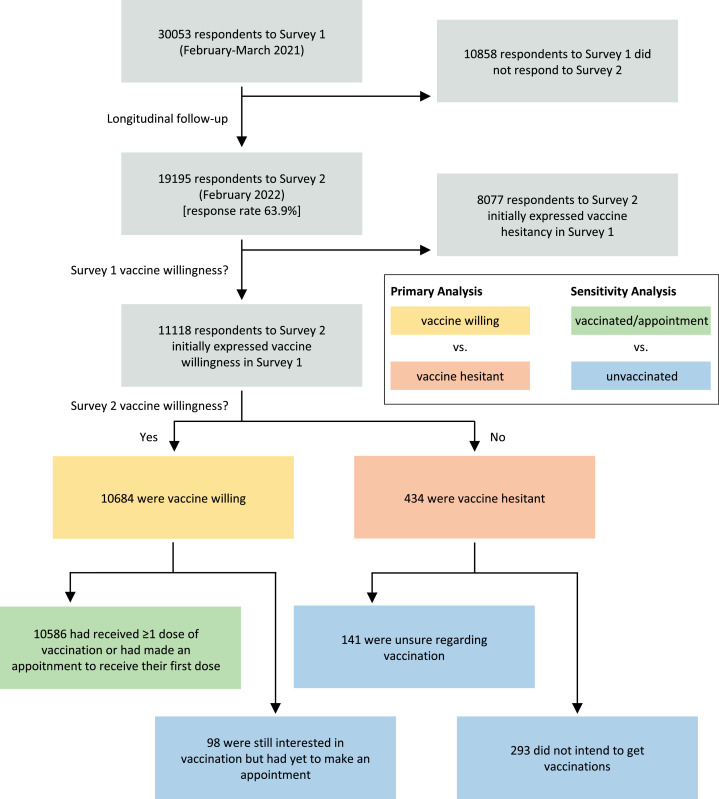
Flowchart of vaccine willingness and hesitancy among Survey 1 and 2 respondents.

Of 19,159 respondents to the second survey, 11,118 (57·9%) had previously expressed interest in being vaccinated in the first survey (Figure 1). Of those respondents, 10,684 (96·1%) and 434 (3·9%) were in the vaccine willing and hesitant groups, respectively. Among the hesitant group, 141 (32·5%) and 293 (67·5%) responded that they are unsure regarding vaccination or do not intend to get vaccinated, respectively.

The socioeconomic characteristics of those initially willing to receive a COVID-19 vaccine (Survey 1), stratified by whether they had developed vaccine hesitancy one year later (Survey 2), are shown in Table 1. Characteristics for all other variables are shown in STable 1. The willing and hesitant groups were similar with respect to gender, region of residence, household size, and occupation type. The willing group was statistically significantly older than the hesitant group (mean age [SE] 58·0 [0·14] vs 48·2 [0·74]). Those in the willing group were more likely to be married (70·6 vs 53·9%), have an undergraduate education or higher (48·0 vs 41·0%), and have an annual income of at least three million yen (74·8 vs 68·1%) than those in the hesitant group.

**Table 1:** Demographic characteristics of survey respondents with vaccine willingness in Survey 1, stratified by vaccine willingness in Survey 2.

	Vaccination Intention		p	Total
	Willing (*N* = 10,684)	Hesitant (*N* = 434)		(*N* = 11,118)
**Gender**				
Women	4841 (45.3)	183 (42.2)	0.347	5024 (45.2)
Men	5833 (54.6)	251 (57.8)		6084 (54.7)
Other	10 (0.1)	0 (0.0)		10 (0.1)
**Age**	58.0 (0.14)	48.2 (0.74)	<0.001	57.6 (0.14)
**Region of Residence**				
Hokkaido & Tohoku	1238 (11.6)	51 (11.8)	0.382	1289 (11.6)
Kanto	3633 (34.0)	141 (32.5)		3774 (33.9)
Chubu	1844 (17.3)	75 (17.3)		1919 (17.3)
Kansai	1857 (17.4)	67 (15.4)		1924 (17.3)
Chugoku & Shikoku	981 (9.2)	40 (9.2)		1021 (9.2)
Kyushu & Okinawa	1131 (10.6)	60 (13.8)		1191 (10.7)
**Marital Status**				
Married	7547 (70.6)	234 (53.9)	<0.001	7781 (70.0)
Unmarried	3137 (29.4)	200 (46.1)		3337 (30.0)
Household Size**	2.5 (0.01)	2.6 (0.06)	0.824	2.5 (0.01)
**Education**				
High school or less	3659 (34.3)	171 (39.4)	0.017	3830 (34.5)
Short college or vocational school	1901 (17.8)	85 (19.6)		1986 (17.9)
Undergraduate studies	4637 (43.4)	155 (35.7)		4792 (43.1)
Graduate studies	487 (4.6)	23 (5.3)		510 (4.6)
**Occupation Type**				
Healthcare workers	737 (6.9)	31 (7.1)	0.57	768 (6.9)
Social & education workers	775 (7.3)	34 (7.8)		809 (7.3)
Other essential workers	3484 (32.6)	154 (35.5)		3638 (32.7)
Non-essential workers	3447 (32.3)	136 (31.3)		3583 (32.2)
Other	2241 (21.0)	79 (18.2)		2320 (20.9)
**Annual Household Income (10,000 JPY)**				
Less than 100	474 (4.4)	41 (9.5)	<0.001	515 (4.6)
100 to 299	2215 (20.7)	97 (22.4)		2312 (20.8)
300 to 499	3225 (30.2)	113 (26.0)		3338 (30.0)
500 to 799	2664 (24.9)	103 (23.7)		2767 (24.9)
800 or more	2106 (19.7)	80 (18.4)		2186 (19.7)

*Household size includes the respondent and is capped at ‘6 or more.’

All categorical variables show N (%); continuous variables (age and household size) show mean (SE).

### Regression analysis

The covariates selected by the MCP-penalized group regression and the results of the subsequent logistic regression are shown in Table 2. Marital status was the only socioeconomic variable selected to be included in the model: married persons were significantly less likely to become vaccine hesitant than their unmarried counterparts (OR [95% CI] 0·72 [0·56-0·92]).

**Table 2:** Odds ratios (95% confidence intervals) for vaccine hesitancy among those with initial vaccine willingness.

	Odds Ratio (95% CI)	p
**Socioeconomic**		
**Marital Status**		
Unmarried	Ref.	
Married	0.72 (0.56 to 0.92)	0.009
**Health**		
**Self-reported health status**		
Very good	Ref.	
Good	1.04 (0.75 to 1.44)	0.829
Fair	0.95 (0.69 to 1.32)	0.773
Poor	1.60 (1.05 to 2.45)	0.029
Very poor	2.14 (1.06 to 4.32)	0.035
**Do you receive influenza vaccinations?**		
Every year	Ref.	
Every few years	1.44 (1.04 to 1.98)	0.027
Rarely or never	1.90 (1.44 to 2.50)	<0.001
**What is your best guess as to whether you will get COVID-19 within the next 6 months?**		
I don't think I will get COVID-19	Ref.	
I think I will get a mild case of COVID-19	0.61 (0.46 to 0.79)	<0.001
I think I will get seriously ill from COVID-19	0.95 (0.65 to 1.40)	0.806
I have already had COVID-19	2.16 (1.16 to 4.01)	0.015
**Have you ever received a COVID-19 test?**		
Yes	Ref.	
No	1.45 (1.09 to 1.92)	0.011
**Do you engage in preventive measures against COVID-19 (e.g., masking, minimizing outings, etc.)?**		
Yes	Ref.	
No	2.35 (1.55 to 3.57)	<0.001
**Psychology and attitudes**		
**How do you feel are the benefits of the COVID-19 vaccine? (perceived benefits of the COVID-19 vaccine)**		
Very small	Ref.	
Small	0.51 (0.26 to 0.99)	0.047
Medium	0.47 (0.25 to 0.89)	0.020
Large	0.20 (0.10 to 0.38)	<0.001
Very large	0.26 (0.12 to 0.55)	<0.001
**How do you think the disadvantages of the COVID-19 vaccine are? (perceived risks of the COVID-19 vaccine)**		
Very small	Ref.	
Small	1.04 (0.52 to 2.06)	0.917
Medium	2.31 (1.19 to 4.49)	0.014
Large	3.87 (1.97 to 7.61)	<0.001
Very large	4.36 (2.06 to 9.21)	<0.001
**If others have been vaccinated against COVID-19, I believe I should be vaccinated as well.**		
Strongly disagree	Ref.	
Disagree	0.55 (0.32 to 0.94)	0.028
Neither agree nor disagree	0.24 (0.14 to 0.39)	<0.001
Agree	0.07 (0.04 to 0.13)	<0.001
Strongly agree	0.08 (0.04 to 0.15)	<0.001
**If you have already been vaccinated, how many people around you were vaccinated at the time you received the first dose, and if not, how many people around you are currently vaccinated?**		
About 0%	Ref.	
About 25%	0.41 (0.24 to 0.70)	0.001
About 50%	0.63 (0.39 to 1.00)	0.052
About 75%	0.55 (0.35 to 0.86)	0.009
About 100%	0.35 (0.21 to 0.57)	<0.001
**For which professions do you believe vaccination should be prioritized? (Multiple Answer)**		
Office workers	0.72 (0.54 to 0.96)	0.025
Medical care	0.48 (0.37 to 0.63)	<0.001
**Do you support or oppose changing various activity restrictions depending on vaccination status (or whether or not one has proof of negative testing)?**		
Support	Ref.	
Neither support nor oppose	1.59 (1.17 to 2.16)	0.003
Oppose	2.69 (1.87 to 3.86)	<0.001
**Which of the following would apply to you if the COVID-19 vaccination were made available to children under 12 years of age in the future?**		
For (have children in the specified age range)	Ref.	
For (do not have children in the specified age range)	1.07 (0.59 to 1.93)	0.826
Neither for nor against (have children in the specified age range)	2.38 (1.19 to 4.76)	0.014
Neither for nor against (do not have children in the specified age range)	1.78 (1.01 to 3.12)	0.046
Against (have children in the specified age range)	3.63 (1.78 to 7.41)	<0.001
Against (do not have children in the specified age range)	1.76 (0.90 to 3.41)	0.096
**Information sources**		
**From what sources do you receive information about COVID-19? (Multiple Answer)**		
*Pharmacists*	0.40 (0.17 to 0.94)	0.035
*Newspapers*	0.62 (0.45 to 0.86)	0.004
*Magazines*	2.03 (1.11 to 3.70)	0.022
*Twitter*	0.56 (0.34 to 0.92)	0.022
*YouTube*	1.69 (1.02 to 2.81)	0.044
*TikTok*	2.05 (0.58 to 7.26)	0.266
*Local authorities such as prefectures and municipalities*	0.54 (0.39 to 0.74)	<0.001
*Other companies*	3.40 (2.10 to 5.51)	<0.001
**How much do you trust information about COVID-19 from the following sources? (4-point scale)**		
*Scientific literature*	0.63 (0.51 to 0.78)	<0.001
*YouTube*	1.21 (0.98 to 1.50)	0.081
*Pharmaceutical companies*	1.18 (0.89 to 1.55)	0.245
*Other companies*	1.41 (1.06 to 1.88)	0.020
**To what extent did you consult information from the following sources in making your decision to vaccinate against COVID-19? (4-point scale)**		
*Facebook*	1.24 (1.05 to 1.46)	0.012

The reference categories for multiple answer questions were those who did not select any given answer choice.

Five health-related variables were found to be significantly associated with developing vaccine hesitancy despite prior vaccine willingness. Those who reported poor health status (Ref = ‘very good’; ‘very poor’ 2·14 [1·06-4·32]), do not receive yearly influenza vaccinations (Ref = ‘every year’; ‘rarely or never’ 1·90 [1·44-2·50]), have already had COVID-19 (2·16 [1·16-4·01]), have never received a COVID-19 test (1·45 [1·09-1·92]), and do not engage in preventive measures against COVID-19 (e.g., masking or minimizing outings) (2·35 [1·55-3·57]) were significantly more likely to report vaccine hesitancy at one-year follow-up.

Eight psychological and attitudinal variables were also selected to be included in the model. Those who believe the benefits of the vaccine to be very small (Ref = ‘very small’; ‘very large’ 0·26 [0·12-0·55]), believe the disadvantages of the vaccine to be intermediate to very large (Ref = ‘very small; ‘very large’ 4·36 [2·06-9·21]), do not believe that they should be vaccinated even if others have (Ref = ‘strongly disagree’; ‘strongly agree’ 0·08 [0·04-0·15]), are surrounded by a low proportion of vaccinated individuals (Ref = ‘0%’; ‘100%’ 0·35 [0·21-0·57]), do not believe office workers (0·72 [0·54-0·96]) or medical professionals (0·48 [0·37-0·63]) should be prioritized for vaccination, do not believe in changing activity restrictions based on vaccination status (Ref = ‘support’; ‘oppose’ 2·69 [1·87-3·86]), and are against vaccinating children under 12 years of age if they have children in that age range (Ref = ‘for and with children in that age range’; ‘against and with children in that age range’ 3·63 [1·78-7·41]) were significantly more likely to report vaccine hesitancy at one-year follow-up.

Thirteen variables concerning sources of COVID-19 information were also selected to be included in the model. Those who receive information from pharmacists (0·40 [0·17-0·94]), newspapers (0·62 [0·45-0·86]), Twitter (0·56 [0·34-0·92]), and local authorities (e.g., prefectures and municipalities) (0·54 [0·39-0·74]) were more likely to remain vaccine willing at one-year follow-up while those who receive information from magazines (2·03 [1·11-3·70]), YouTube (1·69 [1·02-2·81]), TikTok (2·05 [0·58-7·26]), and other companies (3·40 [2·10-5·51]) were more likely to have become vaccine hesitant. Those with lower trust in scientific literature (0·63 [0·51-0·78]) and higher trust in YouTube (1·21 [0·98-1·50]), pharmaceutical companies (1·18 [0·89-1·55]), and other companies (1·41 [1·06-1·88]) were more likely to have become vaccine hesitant. Finally, those who consult Facebook for vaccine-related information to a higher degree (1·24 [1·05-1·46]) were more likely to have become vaccine hesitant.

### Sensitivity analysis

The results of the descriptive analysis and logistic regression with sparse group minimax concave penalty using vaccination status as the binary outcome variable are shown in STables 2-4. In total, 25 covariates were included the model, compared to 27 in the primary analysis. The results largely reflect those of the primary analysis, both in terms of the covariates selected (23 covariates and 43 variables in common) and the direction of their associations. Two variables were selected for inclusion in the sensitivity analysis that were not captured by the primary analysis: lack of confidence filling out medical forms (Ref = ‘not at all confident’; ‘extremely confident’ 0·46 [0·22-0·93]) and the belief that information from dentists is sufficiently disseminated (1·21 [1·06-1·38]) were both associated with a higher likelihood of remaining unvaccinated. Unlike the primary analysis, self-reported health status, use of TikTok for COVID-19 information, trust in other companies for COVID-19 information, and use of Facebook for vaccine-related information were not found to be meaningful predictors of the outcome variable.

## Discussion

Though many studies have attempted to assess reasons for vaccine hesitancy, few have attempted to characterize reasons for vaccine intention reversal, and fewer still have highlighted those who were initially willing to receive vaccines but ultimately became hesitant. In this study, we used longitudinal data from a large, national sample of Japanese adults to characterize those who initially expressed COVID-19 vaccine willingness but reported vaccine hesitancy one year later. We found that several socioeconomic, health-, psychological and attitudinal, and information-related variables were associated with reversal of vaccine willingness.

We found that of those initially expressing desire to be vaccinated, 3·9% became vaccine hesitant in the following year (4·8% remained unvaccinated). A US study found that between 6-19% of those previously willing to vaccinate had ultimately not been inoculated by follow-up nine months later; the proportion varied by race/ethnicity, with Asians (6·0%) being least likely and non-Hispanic Blacks (19·0%) most likely to have changes of heart.^25^ In another nationally representative sample of US adults, 37·3% and 17·0% of those previously expressing interest in vaccination had continued interest in vaccination or had become unsure/unwilling, respectively; however, the follow-up period was less than two months.^24^ A study of patients living with multiple sclerosis in the UK found that 53·2% of those previously desiring vaccination ultimately were inoculated by follow-up at five months, though interpretation is limited by considerable non-response.^22^ A study of refugees living in the US and presenting for COVID-19 testing found that 19·3% of those initially desiring vaccination changed their mind by follow-up four months later.^20^ Our findings are comparable to the Asian-specific proportion reported by Rane et al. (2021) (4·8% vs 6·0%),^25^ whereas short follow-up periods and studies of limited, non-nationally representative subpopulations limit reasonable comparisons with the remaining research. That our findings resemble those in the US Asian population suggest that underlying cultural factors may play a role in determining the propensity to reverse vaccine willingness.

We used group regression with an MCP penalty to select variables that significantly predict vaccine hesitancy despite previous willingness while also encouraging model sparsity. We found that marital status (unmarried), but not other socioeconomic variables, was a significant predictor of reversals of vaccine willingness. Being unmarried/living alone has previously been shown to be associated with increased likelihood of vaccine hesitancy in Japan,^15^ which may be attributable to the desire to protect one's spouse, prevent chains of intra-household transmission (especially among those with children), or conform to a partner's vaccine preferences. Notably, socioeconomic variables such as age, education, occupation, and household income were not selected for inclusion in the model despite statistically significant differences with respect to these covariates between the willing and hesitant groups (Table 1). These variables did not improve the predictive accuracy of the logistic model enough to compensate for the penalty of adding more covariates into the model, suggesting that the group regression with MCP penalty was successful in discerning not only significant but also meaningful predictors of the outcome variable. Though older age has often been associated with increased likelihood of persistent vaccine willingness,^16^^,^^23^ at least one study has found non-linear associations between age and vaccination outcomes among those with prior willingness.^24^ The relationship between education and vaccine hesitancy in Japan is mixed,^14^^,^^15^ but it has not previously been identified as a significant predictor of willingness reversals.^23^^,^^24^ Similarly, the relationship between income and vaccine hesitancy in Japan is also mixed,^14^^,^^15^ and it has not yet been specifically assessed among those who were initially COVID-19 vaccine willing but ultimately did not get inoculated.

We identified five health-related covariates that were significant predictors of remaining vaccine willing at one-year follow-up, including good health status, a history of routinely receiving yearly influenza vaccinations, no history of COVID-19 infection, a history of having received a COVID-19 test, and engagement in preventive measures against COVID-19. Prior research assessing vaccine hesitancy among young Japanese men and women has found that poor health status is a predictor of vaccine hesitancy,^29^ in line with our findings that suggest poor health status may predict reversals of vaccine willingness. However, having comorbidities was found to predict vaccine uptake in a US cohort, suggesting that associations may be mixed.^25^ Consistent with our findings, a history of annual influenza vaccine inoculations has generally been found to predict vaccine uptake and persistent vaccine willingness,^16^^,^^22^ though one study has found evidence to support the opposite conclusion among a population of those living with multiple sclerosis.^23^ Those with annual influenza vaccine routines may have stronger faith in the benefits of vaccines, predisposing them to remain vaccine willing in the face of misinformation and mass reporting of adverse effects. Among those who have recovered from severe COVID-19 infection, the belief that one was now immune to reinfection was associated with reversal of vaccine willingness,^21^ consistent with our findings. Notably, a history of having been tested and engagement in preventive measures against COVID-19 were not identified as significant predictors of vaccine hesitancy in the first round of this survey.^14^ One might hypothesize that those who had been tested or continued to engage in preventive behaviors throughout the course of the pandemic were similarly more likely to pursue other prophylactic measures (i.e., vaccination) compared to those who did not seek out testing or conform to masking guidelines.

We identified eight psychological and attitudinal covariates that were significant predictors of remaining vaccine willing at one-year follow-up, including the belief that one ought to be vaccinated if others are, a high proportion of those in one's vicinity who are vaccinated, a low estimation of the disadvantages and a high estimation of the benefits of the COVID-19 vaccine, which professions one believes should be prioritized for vaccination (office workers and medical professionals), the belief that activity restrictions should be changed based on vaccination status, and the belief that children under 12 years of age should be vaccinated. Our findings suggest that the higher the proportion of those in one's vicinity who are vaccinated, the lower the likelihood of becoming vaccine hesitant by the time of follow-up, which is consistent with other studies that have found reversals of vaccine hesitancy to be associated with knowing friends and family members who have been vaccinated and did not experience adverse effects.^21^ Prior investigations of the determinants of vaccine hesitancy in Japan have found that a sense of collective responsibility and adherence to subjective norms were associated with a lower likelihood of being unsure or unwilling to receive a vaccine,^7^^,^^30^ consistent with a culture that places a high premium on conformity. Those who were initially willing to receive vaccination despite believing there to be many disadvantages or few advantages were more easily swayed to hesitancy, suggesting that their initial willingness may have been tenuous. Those who oppose so-called vaccine passports were more likely to develop vaccine hesitancy, consistent with prior research showing that the topic of activity restrictions based on vaccine status is very polarizing and tends to dissuade those who have vaccine concerns from getting inoculated.^31^ This polarizing effect also appears to be at play in the question regarding vaccination of children under age 12, which may be a particularly sensitive topic for parents.

We identified thirteen information-related covariates that were significant predictors of remaining vaccine willing at one-year follow-up, including the sources from which one receives their information (pharmacists, newspapers, Twitter, and local authorities vs not magazines, YouTube, TikTok, and other companies), how much trust they put in those sources (high trust in scientific literature and low trust in YouTube, pharmaceutical companies, and other companies), and whether they consult sources for vaccine-related information (Facebook). Over the course of the pandemic, misinformation has played a large role in promoting vaccine hesitancy, and likely was responsible for reversals of vaccine willingness in the case of our sample population.^9^^,^^10^ The use of and high trust in YouTube were previously found to be associated with vaccine hesitancy in Japan,^14^ consistent with our findings in which they are associated with reversals of vaccine willingness at one year follow-up. Much of the information disseminated via YouTube is unverified, and vaccine misinformation is notoriously abundant. Contrastingly, the use of Twitter was associated with remaining vaccine willing. There is a strong medical and public health presence on Twitter (e.g., #MedTwitter), and this community was broadened during the pandemic. Thus, though Twitter suffers from a certain degree of misinformation much like other social media, there was also a significant amount of accurate vaccine-related information available, which may explain these seemingly contradictory findings. The same analogy likely applies to magazines and newspapers: magazines tend to be more sensationalized and prone to misinformation than newspapers, and public health messaging may target newspapers more than magazines.

Notably, the use of information from local authorities, such as prefectures and municipalities, was associated with a lower likelihood of remaining unvaccinated. Similar studies have reported that high trust in governmental institutions is associated with fewer changes of heart in previously willing populations.^23^ Similarly, those who place high trust in scientific literature were also less likely to remain unvaccinated, likely because published studies have overwhelmingly supported the individual- and population-level benefits of COVID-19 vaccination. For the same reasoning, trust in pharmacists may prove protective, as pharmacists are likely up to date on the scientific literature. Paradoxically, trust in pharmaceutical companies is associated with vaccine hesitancy; though the reasons behind this trend are unclear, it is possible that those who trust pharmaceutical companies have a higher trust in treatment rather than prevention, and thus forego prophylaxis in favor of therapeutic drugs.

Though it started slow, Japan's vaccination campaign ultimately surpassed 70% vaccination for the two-dose series. Despite relative success in controlling COVID-19 case counts, Japan experienced a renewed wave of infections in February 2022, its largest to date, suggesting that there is still room for improvement. COVID-19 vaccination campaigns stand to gain from targeting those who were initially planning to receive the vaccine but ultimately decided against getting inoculated, as these individuals have previously demonstrated vaccine willingness. Using the characterization of this subpopulation as detailed in this study, public health messaging campaigns should consider how best to recruit this group of individuals, in order to tip the scales in favor of herd immunity.

### Limitations

The survey data used in this study have limitations, which are reported in detail elsewhere.^14^ First, respondents were recruited using an online survey platform that offered points, which can be redeemed to purchase goods or services in return for answering questionnaires. Thus, the representativeness of our sample is subject to self-selection bias, though the use of a quota sampling method based on age, gender, and prefecture yielded a fairly representative sample in the first iteration of this survey, with the main exception being that survey respondents were slightly more educated than the general population of Japan. Second, the follow-up survey did not ask for reasons why respondents may have become vaccine hesitant despite previously being willing in the prior survey. However, this research still constitutes the first step in characterizing this unique subpopulation.

## Conclusions

Individuals who previously expressed willingness to vaccinate but ultimately developed vaccine hesitancy represent an overlooked subpopulation in the vaccine hesitancy literature and in vaccination campaigns, which often focus on those who were unsure or unwilling from the very beginning. This study provides new information regarding the characteristics of those who experienced reversals in vaccine willingness. Our findings shed light on which information sources should be targeted when reaching out to this group of individuals, while also detailing the health-related, psychologic, and attitudinal characteristics of those who should be convinced to follow-through on their original vaccine intentions. Vaccination campaigns require a concerted effort to recruit as many individuals as possible, and the results of this study will serve as a launchpad for accessing those who initially expressed willingness but ultimately became vaccine hesitant.

## Contributors

Conception/design of the work: C.G., D.Y., T.K., and S.N.; acquisition of data: K.M.S., H.K., S.K., K.S., H.M., and S.N.; data curation: C.G., A.E.; analysis of data: C.G.; interpretation of findings: all authors; drafting of the work: C.G.; substantially revised the work: all authors.

## Data sharing statement

The datasets generated during and/or analyzed during the current study are not publicly available due to ethical considerations but are available from the corresponding author on reasonable request.

## Ethics statement

Ethical approval was granted by the Ethics Committee of Keio University School of Medicine under authorization number 20200340. Respondents had to provide their consent before they proceeded to the questionnaire response page.

## Declaration of interests

Arata Takahashi and Hiroaki Miyata are affiliated with the Department of Healthcare Quality Assessment at The University of Tokyo. The department is a social collaboration department supported by grants from the National Clinical Database, Johnson & Johnson K.K., and Nipro Co. The remaining authors declare no conflicts of interest for this article.

## Appendix Supplementary materials

Image, application 1
