# Supplementary material for: Factors associated with reversals of COVID-19 vaccination willingness: Results from two longitudinal, national surveys in Japan 2021-2022
Source: Lancet Reg Health West Pac. 2022 Jul 21;27:100540. doi: 10.1016/j.lanwpc.2022.100540 (PMC9302915; doi:10.1016/j.lanwpc.2022.100540)
Supplement: Supplementary file 1 [file mmc1.docx]

**Online Supplementary Material**

**Table of Contents**

| **Table/Figure** | **Title** | **Page** |
| --- | --- | --- |
| NA | Supplemental Methods | 2-3 |
| STable 1 | Additional characteristics of survey respondents with vaccine willingness in Survey 1, stratified by vaccine willingness in Survey 2. | 4-13 |
| STable 2 | Demographic characteristics of survey respondents with vaccine willingness in Survey 1, stratified by vaccination status at Survey 2. | 14-15 |
| Stable 3 | Additional characteristics of survey respondents with vaccine willingness in Survey 1, stratified by vaccination status at Survey 2. | 16-25 |
| Stable 4 | Odds ratios (95% confidence intervals) for remaining unvaccinated among those with initial vaccine willingness. | 26-28 |
| NA | References | 29 |

**Supplemental Methods**

The following variables were reclassified for the purposes of reducing variable burden:

| **Variable/Question** | **Original Categories** | **Reclassification** |
| --- | --- | --- |
| Prefecture of residence | 47 prefectures (1 to 47) | 6 regions: Hokkaido & Tohoku (1-7), Kanto (8-14), Chubu (15-23), Kansai (24-30), Chugoku & Shikoku (31-39), Kyushu & Okinawa (40-47) |
| Education | Middle school; High school/Technical college; Junior college/Vocational school; University; Master's degree; Graduate school (i.e., PhD) | High school or less; Junior college/Vocational school; Undergraduate; Graduate |
| Occupation | Agriculture, forestry, and fisheries; Construction; Manufacturing; Information and communications; Transportation and postal services; Wholesale and retail trade; Finance and insurance; Real estate and good rental and leasing; Scientific research, professional, and technical services; Accommodations, food and beverage services; Living-related and personal services and amusement services; Education and learning support; Healthcare and welfare; Combines services; Services (not elsewhere classified); Public service (not elsewhere classified); Students; Homemaker; Other | Healthcare workers (Healthcare and welfare); Social and education workers (Accommodations, food and beverage services; Living-related and personal services and amusement services; Education and learning support; Students); Other essential workers (Agriculture, forestry, and fisheries; Construction; Manufacturing; Information and communications; Transportation and postal services; Wholesale and retail trade; Finance and insurance; Real estate and good rental and leasing; Scientific research, professional, and technical services; Public service (not elsewhere classified)); Non-essential workers (Combines services; Services (not elsewhere classified); Homemaker); Other (Other)  *These classifications were based on prior research^1^ and governmental categorizations^2^ |
| Annual household income (10,000 JPY) | Less than 100; 100-199; 200-299; 300-399; 400-499; 500-599; 600-699; 700-799; 800-899; 900-999; 1000 or more | Less than 100; 100-299; 300-499; 500-799; 800 or more |
| Marital status | Married (including de facto marriage); Never married (without a partner); Never married (with a partner); Widowed; Divorced | Married; Unmarried |
| Have you ever received a COVID-19 test? (Multiple Answer) | No; Yes, PCR test; Yes, antigen test; Yes, antibody test; Yes, unsure what kind | No; Yes |
| Do you engage in preventive measures against COVID-19 (e.g., masking, minimizing outings, etc.)? (multiple answer) | Avoid places with poor ventilation; Avoid densely packed places; Avoid speaking when nearby others; Wash hands, gargle, and/or sanitize hands with alcohol; Cover mouth when coughing/sneezing; Telework; Social distancing; Wear a mask when out; Avoid dinners/parties with many guests; Avoiding meals with non-household members; Ventilation; Minimize outings; Other; None in particular | No; Yes |
| Have any family, friends, or colleagues been infected with COVID-19? (multiple answer) | Family (mild illness); Family (moderate illness); Family (severe illness); Family (unsure of severity); Friends and/or colleagues (mild illness); Friends and/or colleagues (moderate illness); Friends and/or colleagues (severe illness); Friends and/or colleagues (unsure of severity); None/Unsure | No; Yes |
| Have you ever refrained from visiting a medical institution because you were anxious about being infected by the new coronavirus? (multiple answer) | Yes, for regular visits; Yes, for sudden symptoms such as cold, headache, abdominal pain, etc.; Yes, for routine physicals and/or cancer screening; Yes, for other appointments; No | No; Yes |

The following variables were considered redundant with several of the other questions assessing information sources and one’s trust in them, so they were not included in the current analysis.

| **Variable/Question** | **Answer options** |
| --- | --- |
| Do you trust scientists in the field of vaccine development for COVID-19? | 5-point Likert scale |
| Do you trust the public authorities who approve vaccines for COVID-19? |  |
| Do you trust your healthcare provider about vaccination against COVID-19? |  |

**STable 1. Additional characteristics of survey respondents with vaccine willingness in Survey 1, stratified by vaccine willingness in Survey 2.**

|  | **Vaccination Intention** | | **p** | **Total** |
| --- | --- | --- | --- | --- |
|  | **Willing**  **(N = 10,684)** | **Hesitant**  **(N = 434)** |  | **(N = 11,118)** |
| **Health** |  |  |  |  |
| **Have you ever received a COVID-19 test?** |  |  |  |  |
| Yes | 2,782 (26.0) | 110 (25.4) | 0.747 | 2,892 (26.0) |
| No | 7,902 (74.0) | 324 (74.7) |  | 8,226 (74.0) |
| **Do you engage in preventive measures against COVID-19 (e.g., masking, minimizing outings, etc.)?** |  |  |  |  |
| Yes | 10,528 (98.5) | 365 (84.1) | <0.001 | 10,893 (98.0) |
| No | 156 (1.5) | 69 (15.9) |  | 225 (2.0) |
| **Have any family, friends, or colleagues been infected with COVID-19?** |  |  |  |  |
| Yes | 2,517 (23.6) | 84 (19.4) | 0.043 | 2,601 (23.4) |
| No or unsure | 8,167 (76.4) | 350 (80.7) |  | 8,517 (76.6) |
| **Have you ever refrained from visiting a medical institution because you were anxious about being infected by the new coronavirus?** |  |  |  |  |
| Yes | 3,698 (34.6) | 138 (31.8) | 0.226 | 3,836 (34.5) |
| No | 6,986 (65.4) | 296 (68.2) |  | 7,282 (65.5) |
| **Presence of underlying diseases (e.g., diabetes, heart failure, respiratory disease [such as COPD]) or use of dialysis, immunosuppressive drugs, or anticancer therapies** |  |  |  |  |
| Yes | 1,778 (16.6) | 44 (10.1) | <0.001 | 1,822 (16.4) |
| No | 8,906 (83.4) | 390 (89.9) |  | 9,296 (83.6) |
| **Living with family members who are elderly or have underlying diseases** |  |  |  |  |
| Yes | 1,986 (18.6) | 55 (12.7) | 0.002 | 2,041 (18.4) |
| No | 8,698 (81.4) | 379 (87.3) |  | 9,077 (81.6) |
| **Do you receive influenza vaccines?** |  |  |  |  |
| Every year | 5,310 (49.7) | 143 (33.0) | <0.001 | 5,453 (49.1) |
| Every few years | 2,059 (19.3) | 96 (22.1) |  | 2,155 (19.4) |
| Rarely or never | 3,315 (31.0) | 195 (44.9) |  | 3,510 (31.6) |
| **Do you receive routine immunizations?** |  |  |  |  |
| All | 2,961 (27.7) | 79 (18.2) | <0.001 | 3,040 (27.3) |
| Partially | 2,348 (22.0) | 74 (17.1) |  | 2,422 (21.8) |
| None | 3,443 (32.2) | 195 (44.9) |  | 3,638 (32.7) |
| Unsure | 1,932 (18.1) | 86 (19.8) |  | 2,018 (18.2) |
| **Self-reported health status** |  |  |  |  |
| Very good | 2,731 (25.6) | 114 (26.3) | <0.001 | 2,845 (25.6) |
| Good | 3,440 (32.2) | 116 (26.7) |  | 3,556 (32.0) |
| Fair | 3,288 (30.8) | 129 (29.7) |  | 3,417 (30.7) |
| Poor | 1,060 (9.9) | 54 (12.4) |  | 1,114 (10.0) |
| Very poor | 165 (1.5) | 21 (4.8) |  | 186 (1.7) |
| **How confident are you when filling out medical forms by yourself? (health literacy)** |  |  |  |  |
| Not at all | 85 (0.8) | 11 (2.5) | <0.001 | 96 (0.9) |
| A little bit | 335 (3.1) | 27 (6.2) |  | 362 (3.3) |
| Somewhat | 1,668 (15.6) | 136 (31.3) |  | 1,804 (16.2) |
| Fairly | 5,095 (47.7) | 153 (35.3) |  | 5,248 (47.2) |
| Extremely | 3,501 (32.8) | 107 (24.7) |  | 3,608 (32.5) |
| **What is your best guess as to whether you will get COVID-19 within the next 6 months?** |  |  |  |  |
| I don't think I will get COVID-19 | 4,986 (46.7) | 191 (44.0) | <0.001 | 5,177 (46.6) |
| I think I will get a mild case of COVID-19 | 4,558 (42.7) | 155 (35.7) |  | 4,713 (42.4) |
| I think I will get seriously ill from COVID-19 | 1,011 (9.5) | 60 (13.8) |  | 1,071 (9.6) |
| I have already had COVID-19 | 129 (1.2) | 28 (6.5) |  | 157 (1.4) |
| **Psychology & Attitudes** |  |  |  |  |
| **How anxious are you about COVID-19?** |  |  |  |  |
| Not at all anxious | 715 (6.7) | 76 (17.5) | <0.001 | 791 (7.1) |
| Vaguely anxious | 6,823 (63.9) | 222 (51.2) |  | 7,045 (63.4) |
| Have a clear sense of anxiety | 2,587 (24.2) | 89 (20.5) |  | 2,676 (24.1) |
| Feel fear and have anxiety | 559 (5.2) | 47 (10.8) |  | 606 (5.5) |
| **To what extent did the COVID-19 pandemic affect your life within the past year?** |  |  |  |  |
| Not at all | 542 (5.1) | 61 (14.1) | <0.001 | 603 (5.4) |
| Not much | 2,833 (26.5) | 136 (31.3) |  | 2,969 (26.7) |
| Somewhat | 5,714 (53.5) | 179 (41.2) |  | 5,893 (53.0) |
| Quite a lot | 1,595 (14.9) | 58 (13.4) |  | 1,653 (14.9) |
| **If you have already been vaccinated, how many people around you were vaccinated at the time you received the first dose, and if not, how many people around you are currently vaccinated?** |  |  |  |  |
| About 0% | 546 (5.1) | 51 (11.8) | <0.001 | 597 (5.4) |
| About 25% | 1,401 (13.1) | 45 (10.4) |  | 1,446 (13.0) |
| About 50% | 1,739 (16.3) | 116 (26.7) |  | 1,855 (16.7) |
| About 75% | 3,941 (36.9) | 160 (36.9) |  | 4,101 (36.9) |
| About 100% | 3,057 (28.6) | 62 (14.3) |  | 3,119 (28.1) |
| **How do you feel are the benefits of the COVID-19 vaccine? (perceived benefits of the COVID-19 vaccine)** |  |  |  |  |
| Very small | 73 (0.7) | 49 (11.3) | <0.001 | 122 (1.1) |
| Small | 261 (2.4) | 56 (12.9) |  | 317 (2.9) |
| Medium | 1,390 (13.0) | 216 (49.8) |  | 1,606 (14.5) |
| Large | 6,657 (62.3) | 87 (20.1) |  | 6,744 (60.7) |
| Very large | 2,303 (21.6) | 26 (6.0) |  | 2,329 (21.0) |
| **How do you think the disadvantages of the COVID-19 vaccine are? (perceived risks of the COVID-19 vaccine)** |  |  |  |  |
| Very small | 1,595 (14.9) | 18 (4.2) | <0.001 | 1,613 (14.5) |
| Small | 4,912 (46.0) | 46 (10.6) |  | 4,958 (44.6) |
| Medium | 2,940 (27.5) | 225 (51.8) |  | 3,165 (28.5) |
| Large | 1,031 (9.7) | 97 (22.4) |  | 1,128 (10.2) |
| Very large | 206 (1.9) | 48 (11.1) |  | 254 (2.3) |
| **If others have been vaccinated against COVID-19, I believe I should be vaccinated as well** |  |  |  |  |
| Strongly disagree | 113 (1.1) | 64 (14.8) | <0.001 | 177 (1.6) |
| Disagree | 388 (3.6) | 90 (20.7) |  | 478 (4.3) |
| Neither agree nor disagree | 1,677 (15.7) | 188 (43.3) |  | 1,865 (16.8) |
| Agree | 5,468 (51.2) | 64 (14.8) |  | 5,532 (49.8) |
| Strongly agree | 3,038 (28.4) | 28 (6.5) |  | 3,066 (27.6) |
| **Getting the COVID-19 vaccine will ease my anxiety** |  |  |  |  |
| Strongly disagree | 109 (1.0) | 68 (15.7) | <0.001 | 177 (1.6) |
| Disagree | 728 (6.8) | 83 (19.1) |  | 811 (7.3) |
| Neither agree nor disagree | 2,104 (19.7) | 164 (37.8) |  | 2,268 (20.4) |
| Agree | 6,560 (61.4) | 94 (21.7) |  | 6,654 (59.9) |
| Strongly agree | 1,183 (11.1) | 25 (5.8) |  | 1,208 (10.9) |
| **For which professions do you believe vaccination should be prioritized? (Multiple Answer)** |  |  |  |  |
| *Office workers (e.g., clerical, planning, development, etc.)* |  |  |  |  |
| No | 6,236 (58.4) | 347 (80.0) | <0.001 | 6,583 (59.2) |
| Yes | 4,448 (41.6) | 87 (20.1) |  | 4,535 (40.8) |
| *Non-office workers (e.g., sales)* |  |  |  |  |
| No | 3,826 (35.8) | 316 (72.8) | <0.001 | 4,142 (37.3) |
| Yes | 6,858 (64.2) | 118 (27.2) |  | 6,976 (62.8) |
| *Non-office workers (e.g., production, manufacturing, on-site, etc.)* |  |  |  |  |
| No | 5,852 (54.8) | 357 (82.3) | <0.001 | 6,209 (55.9) |
| Yes | 4,832 (45.2) | 77 (17.7) |  | 4,909 (44.2) |
| *Food and beverage provision without entertainment* |  |  |  |  |
| No | 6,206 (58.1) | 371 (85.5) | <0.001 | 6,577 (59.2) |
| Yes | 4,478 (41.9) | 63 (14.5) |  | 4,541 (40.8) |
| *Food and beverage provision with entertainment* |  |  |  |  |
| No | 3,171 (29.7) | 298 (68.7) | <0.001 | 3,469 (31.2) |
| Yes | 7,513 (70.3) | 136 (31.3) |  | 7,649 (68.8) |
| *Education* |  |  |  |  |
| No | 2,922 (27.4) | 308 (71.0) | <0.001 | 3,230 (29.1) |
| Yes | 7,762 (72.7) | 126 (29.0) |  | 7,888 (71.0) |
| *Medical care* |  |  |  |  |
| No | 1,983 (18.6) | 278 (64.1) | <0.001 | 2,261 (20.3) |
| Yes | 8,701 (81.4) | 156 (35.9) |  | 8,857 (79.7) |
| *Nursing and homecare* |  |  |  |  |
| No | 2,266 (21.2) | 273 (62.9) | <0.001 | 2,539 (22.8) |
| Yes | 8,418 (78.8) | 161 (37.1) |  | 8,579 (77.2) |
| *Cab drivers* |  |  |  |  |
| No | 3,596 (33.7) | 320 (73.7) | <0.001 | 3,916 (35.2) |
| Yes | 7,088 (66.3) | 114 (26.3) |  | 7,202 (64.8) |
| *Transportation* |  |  |  |  |
| No | 5,478 (51.3) | 368 (84.8) | <0.001 | 5,846 (52.6) |
| Yes | 5,206 (48.7) | 66 (15.2) |  | 5,272 (47.4) |
| *Retail* |  |  |  |  |
| No | 4,972 (46.5) | 349 (80.4) | <0.001 | 5,321 (47.9) |
| Yes | 5,712 (53.5) | 85 (19.6) |  | 5,797 (52.1) |
| *Lodging and leisure* |  |  |  |  |
| No | 4,083 (38.2) | 328 (75.6) | <0.001 | 4,411 (39.7) |
| Yes | 6,601 (61.8) | 106 (24.4) |  | 6,707 (60.3) |
| *Childcare* |  |  |  |  |
| No | 2,824 (26.4) | 311 (71.7) | <0.001 | 3,135 (28.2) |
| Yes | 7,860 (73.6) | 123 (28.3) |  | 7,983 (71.8) |
| *Hairdessing, beauty, and aesthetics* |  |  |  |  |
| No | 4,068 (38.1) | 324 (74.7) | <0.001 | 4,392 (39.5) |
| Yes | 6,616 (61.9) | 110 (25.4) |  | 6,726 (60.5) |
| *Government offices* |  |  |  |  |
| No | 4,703 (44.0) | 345 (79.5) | <0.001 | 5,048 (45.4) |
| Yes | 5,981 (56.0) | 89 (20.5) |  | 6,070 (54.6) |
| *Other income-earning jobs* |  |  |  |  |
| No | 7,325 (68.6) | 395 (91.0) | <0.001 | 7,720 (69.4) |
| Yes | 3,359 (31.4) | 39 (9.0) |  | 3,398 (30.6) |
| *Students* |  |  |  |  |
| No | 5,542 (51.9) | 368 (84.8) | <0.001 | 5,910 (53.2) |
| Yes | 5,142 (48.1) | 66 (15.2) |  | 5,208 (46.8) |
| *Homemakers* |  |  |  |  |
| No | 7,161 (67.0) | 401 (92.4) | <0.001 | 7,562 (68.0) |
| Yes | 3,523 (33.0) | 33 (7.6) |  | 3,556 (32.0) |
| *Other* |  |  |  |  |
| No | 10,466 (98.0) | 428 (98.6) | 0.339 | 10,894 (98.0) |
| Yes | 218 (2.0) | 6 (1.4) |  | 224 (2.0) |
| *None in particular* |  |  |  |  |
| No | 10,056 (94.1) | 301 (69.4) | <0.001 | 10,357 (93.2) |
| Yes | 628 (5.9) | 133 (30.7) |  | 761 (6.8) |
| **Do you support or oppose changing various activity restrictions depending on vaccination status (or whether or not one has proof of negative testing)?** |  |  |  |  |
| Support | 5,983 (56.0) | 92 (21.2) | <0.001 | 6,075 (54.6) |
| Oppose | 759 (7.1) | 136 (31.3) |  | 895 (8.1) |
| Neither support nor oppose | 3,942 (36.9) | 206 (47.5) |  | 4,148 (37.3) |
| **Which of the following would apply to you if the COVID-19 vaccination were made available to children under 12 years of age in the future?** |  |  |  |  |
| For (have children in the specified age range) | 725 (6.8) | 19 (4.4) | <0.001 | 744 (6.7) |
| For (do not have children in the specified age range) | 5,084 (47.6) | 66 (15.2) |  | 5,150 (46.3) |
| Against (have children in the specified age range) | 128 (1.2) | 31 (7.1) |  | 159 (1.4) |
| Against (do not have children in the specified age range) | 370 (3.5) | 69 (15.9) |  | 439 (4.0) |
| Neither for nor against (have children in the specified age range) | 416 (3.9) | 28 (6.5) |  | 444 (4.0) |
| Neither for nor against (do not have children in the specified age range) | 3,961 (37.1) | 221 (50.9) |  | 4,182 (37.6) |
| **Information sources** |  |  |  |  |
| **From what sources do you receive information about COVID-19? (Multiple Answer)** |  |  |  |  |
| *Physicians* |  |  |  |  |
| No | 8,714 (81.6) | 374 (86.2) | 0.015 | 9,088 (81.7) |
| Yes | 1,970 (18.4) | 60 (13.8) |  | 2,030 (18.3) |
| *Nurses* |  |  |  |  |
| No | 10,065 (94.2) | 415 (95.6) | 0.214 | 10,480 (94.3) |
| Yes | 619 (5.8) | 19 (4.4) |  | 638 (5.7) |
| *Pharmacists* |  |  |  |  |
| No | 10,355 (96.9) | 424 (97.7) | 0.357 | 10,779 (97.0) |
| Yes | 329 (3.1) | 10 (2.3) |  | 339 (3.1) |
| *Veterinarians* |  |  |  |  |
| No | 10,665 (99.8) | 431 (99.3) | 0.018 | 11,096 (99.8) |
| Yes | 19 (0.2) | 3 (0.7) |  | 22 (0.2) |
| *Dentists* |  |  |  |  |
| No | 10,569 (98.9) | 429 (98.9) | 0.881 | 10,998 (98.9) |
| Yes | 115 (1.1) | 5 (1.2) |  | 120 (1.1) |
| *Health fairs & events* |  |  |  |  |
| No | 10,598 (99.2) | 428 (98.6) | 0.193 | 11,026 (99.2) |
| Yes | 86 (0.8) | 6 (1.4) |  | 92 (0.8) |
| *Newspapers* |  |  |  |  |
| No | 5,961 (55.8) | 366 (84.3) | <0.001 | 6,327 (56.9) |
| Yes | 4,723 (44.2) | 68 (15.7) |  | 4,791 (43.1) |
| *Magazines* |  |  |  |  |
| No | 10,113 (94.7) | 415 (95.6) | 0.379 | 10,528 (94.7) |
| Yes | 571 (5.3) | 19 (4.4) |  | 590 (5.3) |
| *Books* |  |  |  |  |
| No | 10,372 (97.1) | 416 (95.9) | 0.14 | 10,788 (97.0) |
| Yes | 312 (2.9) | 18 (4.2) |  | 330 (3.0) |
| *Scientific literature* |  |  |  |  |
| No | 10,540 (98.7) | 427 (98.4) | 0.64 | 10,967 (98.6) |
| Yes | 144 (1.4) | 7 (1.6) |  | 151 (1.4) |
| *Television* |  |  |  |  |
| No | 1,362 (12.8) | 172 (39.6) | <0.001 | 1,534 (13.8) |
| Yes | 9,322 (87.3) | 262 (60.4) |  | 9,584 (86.2) |
| *Radio* |  |  |  |  |
| No | 9,130 (85.5) | 395 (91.0) | 0.001 | 9,525 (85.7) |
| Yes | 1,554 (14.6) | 39 (9.0) |  | 1,593 (14.3) |
| *Internet news sites* |  |  |  |  |
| No | 4,969 (46.5) | 259 (59.7) | <0.001 | 5,228 (47.0) |
| Yes | 5,715 (53.5) | 175 (40.3) |  | 5,890 (53.0) |
| *Search engines (Google, Yahoo, etc.)* |  |  |  |  |
| No | 8,338 (78.0) | 350 (80.7) | 0.198 | 8,688 (78.1) |
| Yes | 2,346 (22.0) | 84 (19.4) |  | 2,430 (21.9) |
| *LINE* |  |  |  |  |
| No | 9,990 (93.5) | 403 (92.9) | 0.592 | 10,393 (93.5) |
| Yes | 694 (6.5) | 31 (7.1) |  | 725 (6.5) |
| *Facebook* |  |  |  |  |
| No | 10,489 (98.2) | 424 (97.7) | 0.467 | 10,913 (98.2) |
| Yes | 195 (1.8) | 10 (2.3) |  | 205 (1.8) |
| *Twitter* |  |  |  |  |
| No | 10,077 (94.3) | 402 (92.6) | 0.138 | 10,479 (94.3) |
| Yes | 607 (5.7) | 32 (7.4) |  | 639 (5.8) |
| *Instagram* |  |  |  |  |
| No | 10,553 (98.8) | 421 (97.0) | 0.001 | 10,974 (98.7) |
| Yes | 131 (1.2) | 13 (3.0) |  | 144 (1.3) |
| *YouTube* |  |  |  |  |
| No | 10,214 (95.6) | 397 (91.5) | <0.001 | 10,611 (95.4) |
| Yes | 470 (4.4) | 37 (8.5) |  | 507 (4.6) |
| *TikTok* |  |  |  |  |
| No | 10,653 (99.7) | 428 (98.6) | <0.001 | 11,081 (99.7) |
| Yes | 31 (0.3) | 6 (1.4) |  | 37 (0.3) |
| *Medical information sites* |  |  |  |  |
| No | 10,370 (97.1) | 424 (97.7) | 0.441 | 10,794 (97.1) |
| Yes | 314 (2.9) | 10 (2.3) |  | 324 (2.9) |
| *Blogs or celebrity web pages* |  |  |  |  |
| No | 10,515 (98.4) | 426 (98.2) | 0.67 | 10,941 (98.4) |
| Yes | 169 (1.6) | 8 (1.8) |  | 177 (1.6) |
| *Local authorities such as prefectures and municipalities* |  |  |  |  |
| No | 6,362 (59.6) | 367 (84.6) | <0.001 | 6,729 (60.5) |
| Yes | 4,322 (40.5) | 67 (15.4) |  | 4,389 (39.5) |
| *Government* |  |  |  |  |
| No | 8,321 (77.9) | 387 (89.2) | <0.001 | 8,708 (78.3) |
| Yes | 2,363 (22.1) | 47 (10.8) |  | 2,410 (21.7) |
| *Medical task forces* |  |  |  |  |
| No | 9,530 (89.2) | 414 (95.4) | <0.001 | 9,944 (89.4) |
| Yes | 1,154 (10.8) | 20 (4.6) |  | 1,174 (10.6) |
| *Friends* |  |  |  |  |
| No | 8,951 (83.8) | 400 (92.2) | <0.001 | 9,351 (84.1) |
| Yes | 1,733 (16.2) | 34 (7.8) |  | 1,767 (15.9) |
| *Family members* |  |  |  |  |
| No | 8,341 (78.1) | 384 (88.5) | <0.001 | 8,725 (78.5) |
| Yes | 2,343 (21.9) | 50 (11.5) |  | 2,393 (21.5) |
| *Scientists and researchers* |  |  |  |  |
| No | 10,343 (96.8) | 422 (97.2) | 0.619 | 10,765 (96.8) |
| Yes | 341 (3.2) | 12 (2.8) |  | 353 (3.2) |
| *Pharmaceutical companies* |  |  |  |  |
| No | 10,541 (98.7) | 430 (99.1) | 0.456 | 10,971 (98.7) |
| Yes | 143 (1.3) | 4 (0.9) |  | 147 (1.3) |
| *Other companies* |  |  |  |  |
| No | 10,536 (98.6) | 384 (88.5) | <0.001 | 10,920 (98.2) |
| Yes | 148 (1.4) | 50 (11.5) |  | 198 (1.8) |
| **How much do you trust information about COVID-19 from the following sources? (4-point scale)** |  |  |  |  |
| Physicians | 2.8 (0.01) | 2.5 (0.04) | <0.001 | 2.8 (0.01) |
| Nurses | 2.7 (0.01) | 2.4 (0.04) | <0.001 | 2.6 (0.01) |
| Pharmacists | 2.5 (0.01) | 2.4 (0.04) | <0.001 | 2.5 (0.01) |
| Veterinarians | 2.1 (0.01) | 2.2 (0.04) | 0.0028 | 2.1 (0.01) |
| Dentists | 2.2 (0.01) | 2.2 (0.04) | 0.6888 | 2.2 (0.01) |
| Health fairs & events | 2.0 (0.01) | 2.1 (0.04) | 0.0303 | 2.0 (0.01) |
| Newspapers | 2.4 (0.01) | 2.1 (0.04) | <0.001 | 2.4 (0.01) |
| Magazines | 2.0 (0.01) | 2.0 (0.04) | 0.5435 | 2.0 (0.01) |
| Books | 2.1 (0.01) | 2.1 (0.04) | 0.951 | 2.1 (0.01) |
| Scientific literature | 2.4 (0.01) | 2.2 (0.03) | <0.001 | 2.4 (0.01) |
| Television | 2.3 (0.01) | 2.2 (0.04) | <0.001 | 2.3 (0.01) |
| Radio | 2.2 (0.01) | 2.1 (0.04) | <0.001 | 2.2 (0.01) |
| Internet news sites | 2.1 (0.01) | 2.1 (0.03) | 0.2972 | 2.1 (0.01) |
| Search engines (Google, Yahoo, etc.) | 2.0 (0.01) | 2.1 (0.04) | 0.0022 | 2.0 (0.01) |
| LINE | 1.7 (0.01) | 1.9 (0.04) | <0.001 | 1.7 (0.01) |
| Facebook | 1.5 (0.01) | 1.8 (0.04) | <0.001 | 1.5 (0.01) |
| Twitter | 1.5 (0.01) | 1.8 (0.04) | <0.001 | 1.5 (0.01) |
| Instagram | 1.5 (0.01) | 1.8 (0.04) | <0.001 | 1.5 (0.01) |
| YouTube | 1.5 (0.01) | 1.9 (0.04) | <0.001 | 1.6 (0.01) |
| TikTok | 1.4 (0.01) | 1.7 (0.04) | <0.001 | 1.4 (0.01) |
| Medical information sites | 2.2 (0.01) | 2.1 (0.04) | 0.0038 | 2.2 (0.01) |
| Blogs or celebrity web pages | 1.6 (0.01) | 1.9 (0.04) | <0.001 | 1.6 (0.01) |
| Local authorities such as prefectures and municipalities | 2.5 (0.01) | 2.2 (0.04) | <0.001 | 2.5 (0.01) |
| Government | 2.4 (0.01) | 2.1 (0.04) | <0.001 | 2.4 (0.01) |
| Medical task forces | 2.5 (0.01) | 2.2 (0.04) | <0.001 | 2.5 (0.01) |
| Friends | 2.0 (0.01) | 2.1 (0.04) | 0.0153 | 2.0 (0.01) |
| Family members | 2.3 (0.01) | 2.2 (0.04) | 0.4725 | 2.3 (0.01) |
| Scientists and researchers | 2.4 (0.01) | 2.2 (0.04) | <0.001 | 2.4 (0.01) |
| Pharmaceutical companies | 2.3 (0.01) | 2.1 (0.04) | 0.0002 | 2.3 (0.01) |
| Other companies | 1.9 (0.01) | 2.0 (0.04) | 0.0053 | 1.9 (0.01) |
| **Do you believe the following COVID-19 information sources are sufficiently disseminated? (4-point scale)** |  |  |  |  |
| Physicians | 2.6 (0.01) | 2.5 (0.04) | 0.0468 | 2.6 (0.01) |
| Nurses | 2.4 (0.01) | 2.5 (0.04) | 0.5235 | 2.4 (0.01) |
| Pharmacists | 2.3 (0.01) | 2.4 (0.04) | 0.1111 | 2.4 (0.01) |
| Veterinarians | 2.1 (0.01) | 2.3 (0.04) | <0.001 | 2.1 (0.01) |
| Dentists | 2.2 (0.01) | 2.4 (0.04) | <0.001 | 2.2 (0.01) |
| Health fairs & events | 2.2 (0.01) | 2.3 (0.04) | 0.0004 | 2.2 (0.01) |
| Newspapers | 2.6 (0.01) | 2.4 (0.04) | <0.001 | 2.6 (0.01) |
| Magazines | 2.2 (0.01) | 2.3 (0.04) | 0.0326 | 2.2 (0.01) |
| Books | 2.3 (0.01) | 2.4 (0.04) | 0.002 | 2.3 (0.01) |
| Scientific literature | 2.5 (0.01) | 2.4 (0.04) | 0.5014 | 2.5 (0.01) |
| Television | 2.7 (0.01) | 2.4 (0.04) | <0.001 | 2.6 (0.01) |
| Radio | 2.5 (0.01) | 2.3 (0.04) | 0.002 | 2.5 (0.01) |
| Internet news sites | 2.4 (0.01) | 2.4 (0.04) | 0.1764 | 2.4 (0.01) |
| Search engines (Google, Yahoo, etc.) | 2.4 (0.01) | 2.4 (0.04) | 0.6829 | 2.4 (0.01) |
| LINE | 2.1 (0.01) | 2.2 (0.04) | 0.0001 | 2.1 (0.01) |
| Facebook | 2.0 (0.01) | 2.2 (0.04) | <0.001 | 2.0 (0.01) |
| Twitter | 2.0 (0.01) | 2.2 (0.04) | <0.001 | 2.0 (0.01) |
| Instagram | 2.0 (0.01) | 2.2 (0.04) | <0.001 | 2.0 (0.01) |
| YouTube | 2.0 (0.01) | 2.2 (0.04) | <0.001 | 2.0 (0.01) |
| TikTok | 1.9 (0.01) | 2.1 (0.04) | <0.001 | 1.9 (0.01) |
| Medical information sites | 2.4 (0.01) | 2.4 (0.04) | 0.4142 | 2.4 (0.01) |
| Blogs or celebrity web pages | 2.1 (0.01) | 2.2 (0.04) | 0.0001 | 2.1 (0.01) |
| Local authorities such as prefectures and municipalities | 2.6 (0.01) | 2.4 (0.04) | <0.001 | 2.6 (0.01) |
| Government | 2.4 (0.01) | 2.3 (0.04) | 0.0041 | 2.4 (0.01) |
| Medical task forces | 2.5 (0.01) | 2.4 (0.04) | <0.001 | 2.5 (0.01) |
| Friends | 2.3 (0.01) | 2.4 (0.04) | 0.0007 | 2.3 (0.01) |
| Family members | 2.4 (0.01) | 2.4 (0.04) | 0.1246 | 2.4 (0.01) |
| Scientists and researchers | 2.4 (0.01) | 2.4 (0.04) | 0.2364 | 2.4 (0.01) |
| Pharmaceutical companies | 2.3 (0.01) | 2.3 (0.04) | 0.9298 | 2.3 (0.01) |
| Other companies | 2.2 (0.01) | 2.3 (0.04) | 0.0015 | 2.2 (0.01) |
| **To what extent did you consult information from the following sources in making your decision to vaccinate against COVID-19? (4-point scale)** |  |  |  |  |
| Physicians | 3.0 (0.01) | 2.7 (0.04) | <0.001 | 3.0 (0.01) |
| Nurses | 2.7 (0.01) | 2.5 (0.04) | <0.001 | 2.7 (0.01) |
| Pharmacists | 2.5 (0.01) | 2.4 (0.04) | 0.0017 | 2.5 (0.01) |
| Veterinarians | 2.0 (0.01) | 2.1 (0.04) | <0.001 | 2.0 (0.01) |
| Dentists | 2.1 (0.01) | 2.2 (0.04) | 0.0264 | 2.1 (0.01) |
| Health fairs & events | 2.1 (0.01) | 2.2 (0.04) | 0.0057 | 2.1 (0.01) |
| Newspapers | 2.6 (0.01) | 2.3 (0.04) | <0.001 | 2.6 (0.01) |
| Magazines | 2.0 (0.01) | 2.1 (0.04) | 0.0474 | 2.0 (0.01) |
| Books | 2.1 (0.01) | 2.2 (0.04) | 0.0127 | 2.1 (0.01) |
| Scientific literature | 2.3 (0.01) | 2.3 (0.04) | 0.5545 | 2.3 (0.01) |
| Television | 2.8 (0.01) | 2.5 (0.04) | <0.001 | 2.8 (0.01) |
| Radio | 2.3 (0.01) | 2.2 (0.04) | 0.0206 | 2.3 (0.01) |
| Internet news sites | 2.4 (0.01) | 2.4 (0.04) | 0.3803 | 2.4 (0.01) |
| Search engines (Google, Yahoo, etc.) | 2.3 (0.01) | 2.3 (0.04) | 0.1473 | 2.3 (0.01) |
| LINE | 1.8 (0.01) | 2.0 (0.04) | <0.001 | 1.8 (0.01) |
| Facebook | 1.7 (0.01) | 2.0 (0.04) | <0.001 | 1.7 (0.01) |
| Twitter | 1.7 (0.01) | 2.0 (0.04) | <0.001 | 1.7 (0.01) |
| Instagram | 1.6 (0.01) | 1.9 (0.04) | <0.001 | 1.6 (0.01) |
| YouTube | 1.7 (0.01) | 2.0 (0.04) | <0.001 | 1.7 (0.01) |
| TikTok | 1.6 (0.01) | 1.9 (0.04) | <0.001 | 1.6 (0.01) |
| Medical information sites | 2.2 (0.01) | 2.2 (0.04) | 0.5485 | 2.2 (0.01) |
| Blogs or celebrity web pages | 1.7 (0.01) | 2.0 (0.04) | <0.001 | 1.8 (0.01) |
| Local authorities such as prefectures and municipalities | 2.8 (0.01) | 2.4 (0.04) | <0.001 | 2.8 (0.01) |
| Government | 2.7 (0.01) | 2.3 (0.04) | <0.001 | 2.7 (0.01) |
| Medical task forces | 2.7 (0.01) | 2.3 (0.04) | <0.001 | 2.7 (0.01) |
| Friends | 2.2 (0.01) | 2.3 (0.04) | 0.1825 | 2.2 (0.01) |
| Family members | 2.5 (0.01) | 2.4 (0.04) | 0.1424 | 2.4 (0.01) |
| Scientists and researchers | 2.4 (0.01) | 2.3 (0.04) | 0.0043 | 2.4 (0.01) |
| Pharmaceutical companies | 2.3 (0.01) | 2.2 (0.04) | 0.0537 | 2.3 (0.01) |
| Other companies | 2.0 (0.01) | 2.1 (0.04) | 0.0029 | 2.0 (0.01) |

**STable 2. Demographic characteristics of survey respondents with vaccine willingness in Survey 1, stratified by vaccination status at Survey 2.**

|  | **Vaccination Status** | | **p** | **Total** |
| --- | --- | --- | --- | --- |
|  | **Vaccinated/**  **Appointment**  **(N = 10,586)** | **Unvaccinated**  **(N = 532)** |  | **(N = 11,118)** |
| **Gender** |  |  |  |  |
| Women | 4,803 (45.4) | 221 (41.5) | 0.171 | 5,024 (45.2) |
| Men | 5,774 (54.5) | 310 (58.3) |  | 6,084 (54.7) |
| Other | 9 (0.1) | 1 (0.2) |  | 10 (0.1) |
|  |  |  |  |  |
| **Age** | 58.1 (0.14) | 48.0 (0.67) | <0.001 | 57.6 (0.14) |
|  |  |  |  |  |
| **Region of Residence** |  |  |  |  |
| Hokkaido & Tohoku | 1,227 (11.6) | 62 (11.7) | 0.343 | 1,289 (11.6) |
| Kanto | 3,607 (34.1) | 167 (31.4) |  | 3,774 (33.9) |
| Chubu | 1,824 (17.2) | 95 (17.9) |  | 1,919 (17.3) |
| Kansai | 1,837 (17.4) | 87 (16.4) |  | 1,924 (17.3) |
| Chugoku & Shikoku | 972 (9.2) | 49 (9.2) |  | 1,021 (9.2) |
| Kyushu & Okinawa | 1,119 (10.6) | 72 (13.5) |  | 1,191 (10.7) |
|  |  |  |  |  |
| **Marital Status** |  |  |  |  |
| Married | 7,502 (70.9) | 279 (52.4) | <0.001 | 7,781 (70.0) |
| Unmarried | 3,084 (29.1) | 253 (47.6) |  | 3,337 (30.0) |
|  |  |  |  |  |
| **Household Size^*^** | 2.5 (0.01) | 2.6 (0.05) | 0.279 | 2.5 (0.01) |
|  |  |  |  |  |
| **Education** |  |  |  |  |
| High school or less | 3,626 (34.3) | 204 (38.4) | 0.018 | 3,830 (34.5) |
| Short college or vocational school | 1,880 (17.8) | 106 (19.9) |  | 1,986 (17.9) |
| Undergraduate studies | 4,598 (43.4) | 194 (36.5) |  | 4,792 (43.1) |
| Graduate studies | 482 (4.6) | 28 (5.3) |  | 510 (4.6) |
|  |  |  |  |  |
| **Occupation Type** |  |  |  |  |
| Healthcare workers | 735 (6.9) | 33 (6.2) | 0.054 | 768 (6.9) |
| Social & education workers | 767 (7.3) | 42 (7.9) |  | 809 (7.3) |
| Other essential workers | 3,435 (32.5) | 203 (38.2) |  | 3,638 (32.6) |
| Non-essential workers | 3,423 (32.3) | 160 (30.1) |  | 3,583 (32.2) |
| Other | 2,226 (21.0) | 94 (17.7) |  | 2,320 (20.9) |
|  |  |  |  |  |
| **Annual Household Income (10,000 JPY)** |  |  |  |  |
| Less than 100 | 465 (4.4) | 50 (9.4) | <0.001 | 515 (4.6) |
| 100 to 299 | 2,189 (20.7) | 123 (23.1) |  | 2,312 (20.8) |
| 300 to 499 | 3,206 (30.3) | 132 (24.8) |  | 3,338 (30.0) |
| 500 to 799 | 2,643 (25.0) | 124 (23.3) |  | 2,767 (24.9) |
| 800 or more | 2,083 (19.7) | 103 (19.4) |  | 2,186 (19.7) |

^*^ Household size includes the respondent and is capped at ‘6 or more.’

All categorical variables show N (%); continuous variables (age and household size) show mean (SE).

**STable 3. Additional characteristics of survey respondents with vaccine willingness in Survey 1, stratified by vaccination status at Survey 2.**

|  | **Vaccination Status** | | **p** | **Total** |
| --- | --- | --- | --- | --- |
|  | **Vaccinated/**  **Appointment**  **(N = 10,586)** | **Unvaccinated**  **(N = 532)** |  | **(N = 11,118)** |
| **Health** |  |  |  |  |
| **Have you ever received a COVID-19 test?** |  |  |  |  |
| Yes | 2,752 (26.0) | 140 (26.3) | 0.87 | 2,892 (26.0) |
| No | 7,834 (74.0) | 392 (73.7) |  | 8,226 (74.0) |
| **Do you engage in preventive measures against COVID-19 (e.g., masking, minimizing outings, etc.)?** |  |  |  |  |
| Yes | 10,435 (98.6) | 458 (86.1) | <0.001 | 10,893 (98.0) |
| No | 151 (1.4) | 74 (13.9) |  | 225 (2.0) |
| **Have any family, friends, or colleagues been infected with COVID-19?** |  |  |  |  |
| Yes | 2,490 (23.5) | 111 (20.9) | 0.158 | 2,601 (23.4) |
| No or unsure | 8,096 (76.5) | 421 (79.1) |  | 8,517 (76.6) |
| **Have you ever refrained from visiting a medical institution because you were anxious about being infected by the new coronavirus?** |  |  |  |  |
| Yes | 3,649 (34.5) | 187 (35.2) | 0.747 | 3,836 (34.5) |
| No | 6,937 (65.5) | 345 (64.9) |  | 7,282 (65.5) |
| **Presence of underlying diseases (e.g., diabetes, heart failure, respiratory disease [such as COPD]) or use of dialysis, immunosuppressive drugs, or anticancer therapies** |  |  |  |  |
| Yes | 1,769 (16.7) | 53 (10.0) | <0.001 | 1,822 (16.4) |
| No | 8,817 (83.3) | 479 (90.0) |  | 9,296 (83.6) |
| **Living with family members who are elderly or have underlying diseases** |  |  |  |  |
| Yes | 1,972 (18.6) | 69 (13.0) | 0.001 | 2,041 (18.4) |
| No | 8,614 (81.4) | 463 (87.0) |  | 9,077 (81.6) |
| **Do you receive influenza vaccines?** |  |  |  |  |
| Every year | 5,279 (49.9) | 174 (32.7) | <0.001 | 5,453 (49.1) |
| Every few years | 2,042 (19.3) | 113 (21.2) |  | 2,155 (19.4) |
| Rarely or never | 3,265 (30.8) | 245 (46.1) |  | 3,510 (31.6) |
| **Do you receive routine immunizations?** |  |  |  |  |
| All | 2,942 (27.8) | 98 (18.4) | <0.001 | 3,040 (27.3) |
| Partially | 2,323 (21.9) | 99 (18.6) |  | 2,422 (21.8) |
| None | 3,396 (32.1) | 242 (45.5) |  | 3,638 (32.7) |
| Unsure | 1,925 (18.2) | 93 (17.5) |  | 2,018 (18.2) |
| **Self-reported health status** |  |  |  |  |
| Very good | 2,707 (25.6) | 138 (25.9) | <0.001 | 2,845 (25.6) |
| Good | 3,421 (32.3) | 135 (25.4) |  | 3,556 (32.0) |
| Fair | 3,249 (30.7) | 168 (31.6) |  | 3,417 (30.7) |
| Poor | 1,046 (9.9) | 68 (12.8) |  | 1,114 (10.0) |
| Very poor | 163 (1.5) | 23 (4.3) |  | 186 (1.7) |
| **How confident are you when filling out medical forms by yourself? (health literacy)** |  |  |  |  |
| Not at all | 81 (0.8) | 15 (2.8) | <0.001 | 96 (0.9) |
| A little bit | 328 (3.1) | 34 (6.4) |  | 362 (3.3) |
| Somewhat | 1,632 (15.4) | 172 (32.3) |  | 1,804 (16.2) |
| Fairly | 5,061 (47.8) | 187 (35.2) |  | 5,248 (47.2) |
| Extremely | 3,484 (32.9) | 124 (23.3) |  | 3,608 (32.5) |
| **What is your best guess as to whether you will get COVID-19 within the next 6 months?** |  |  |  |  |
| I don't think I will get COVID-19 | 4,961 (46.9) | 216 (40.6) | <0.001 | 5,177 (46.6) |
| I think I will get a mild case of COVID-19 | 4,508 (42.6) | 205 (38.5) |  | 4,713 (42.4) |
| I think I will get seriously ill from COVID-19 | 993 (9.4) | 78 (14.7) |  | 1,071 (9.6) |
| I have already had COVID-19 | 124 (1.2) | 33 (6.2) |  | 157 (1.4) |
| **Psychology & Attitudes** |  |  |  |  |
| **How anxious are you about COVID-19?** |  |  |  |  |
| Not at all anxious | 706 (6.7) | 85 (16.0) | <0.001 | 791 (7.1) |
| Vaguely anxious | 6,770 (64.0) | 275 (51.7) |  | 7,045 (63.4) |
| Have a clear sense of anxiety | 2,565 (24.2) | 111 (20.9) |  | 2,676 (24.1) |
| Feel fear and have anxiety | 545 (5.2) | 61 (11.5) |  | 606 (5.5) |
| **To what extent did the COVID-19 pandemic affect your life within the past year?** |  |  |  |  |
| Not at all | 530 (5.0) | 73 (13.7) | <0.001 | 603 (5.4) |
| Not much | 2,805 (26.5) | 164 (30.8) |  | 2,969 (26.7) |
| Somewhat | 5,672 (53.6) | 221 (41.5) |  | 5,893 (53.0) |
| Quite a lot | 1,579 (14.9) | 74 (13.9) |  | 1,653 (14.9) |
| **If you have already been vaccinated, how many people around you were vaccinated at the time you received the first dose, and if not, how many people around you are currently vaccinated?** |  |  |  |  |
| About 0% | 532 (5.0) | 65 (12.2) | <0.001 | 597 (5.4) |
| About 25% | 1,382 (13.1) | 64 (12.0) |  | 1,446 (13.0) |
| About 50% | 1,711 (16.2) | 144 (27.1) |  | 1,855 (16.7) |
| About 75% | 3,913 (37.0) | 188 (35.3) |  | 4,101 (36.9) |
| About 100% | 3,048 (28.8) | 71 (13.4) |  | 3,119 (28.1) |
| **How do you feel are the benefits of the COVID-19 vaccine? (perceived benefits of the COVID-19 vaccine)** |  |  |  |  |
| Very small | 68 (0.6) | 54 (10.2) | <0.001 | 122 (1.1) |
| Small | 250 (2.4) | 67 (12.6) |  | 317 (2.9) |
| Medium | 1,366 (12.9) | 240 (45.1) |  | 1,606 (14.5) |
| Large | 6,610 (62.4) | 134 (25.2) |  | 6,744 (60.7) |
| Very large | 2,292 (21.7) | 37 (7.0) |  | 2,329 (21.0) |
| **How do you think the disadvantages of the COVID-19 vaccine are? (perceived risks of the COVID-19 vaccine)** |  |  |  |  |
| Very small | 1,591 (15.0) | 22 (4.1) | <0.001 | 1,613 (14.5) |
| Small | 4,879 (46.1) | 79 (14.9) |  | 4,958 (44.6) |
| Medium | 2,899 (27.4) | 266 (50.0) |  | 3,165 (28.5) |
| Large | 1,014 (9.6) | 114 (21.4) |  | 1,128 (10.2) |
| Very large | 203 (1.9) | 51 (9.6) |  | 254 (2.3) |
| **If others have been vaccinated against COVID-19, I believe I should be vaccinated as well** |  |  |  |  |
| Strongly disagree | 111 (1.1) | 66 (12.4) | <0.001 | 177 (1.6) |
| Disagree | 380 (3.6) | 98 (18.4) |  | 478 (4.3) |
| Neither agree nor disagree | 1,652 (15.6) | 213 (40.0) |  | 1,865 (16.8) |
| Agree | 5,415 (51.2) | 117 (22.0) |  | 5,532 (49.8) |
| Strongly agree | 3,028 (28.6) | 38 (7.1) |  | 3,066 (27.6) |
| **Getting the COVID-19 vaccine will ease my anxiety** |  |  |  |  |
| Strongly disagree | 107 (1.0) | 70 (13.2) | <0.001 | 177 (1.6) |
| Disagree | 718 (6.8) | 93 (17.5) |  | 811 (7.3) |
| Neither agree nor disagree | 2,073 (19.6) | 195 (36.7) |  | 2,268 (20.4) |
| Agree | 6,512 (61.5) | 142 (26.7) |  | 6,654 (59.9) |
| Strongly agree | 1,176 (11.1) | 32 (6.0) |  | 1,208 (10.9) |
| **For which professions do you believe vaccination should be prioritized? (Multiple Answer)** |  |  |  |  |
| *Office workers (e.g., clerical, planning, development, etc.)* |  |  |  |  |
| No | 6,166 (58.3) | 417 (78.4) | <0.001 | 6,583 (59.2) |
| Yes | 4,420 (41.8) | 115 (21.6) |  | 4,535 (40.8) |
| *Non-office workers (e.g., sales)* |  |  |  |  |
| No | 3,766 (35.6) | 376 (70.7) | <0.001 | 4,142 (37.3) |
| Yes | 6,820 (64.4) | 156 (29.3) |  | 6,976 (62.8) |
| *Non-office workers (e.g., production, manufacturing, on-site, etc.)* |  |  |  |  |
| No | 5,781 (54.6) | 428 (80.5) | <0.001 | 6,209 (55.9) |
| Yes | 4,805 (45.4) | 104 (19.6) |  | 4,909 (44.2) |
| *Food and beverage provision without entertainment* |  |  |  |  |
| No | 6,132 (57.9) | 445 (83.7) | <0.001 | 6,577 (59.2) |
| Yes | 4,454 (42.1) | 87 (16.4) |  | 4,541 (40.8) |
| *Food and beverage provision with entertainment* |  |  |  |  |
| No | 3,121 (29.5) | 348 (65.4) | <0.001 | 3,469 (31.2) |
| Yes | 7,465 (70.5) | 184 (34.6) |  | 7,649 (68.8) |
| *Education* |  |  |  |  |
| No | 2,869 (27.1) | 361 (67.9) | <0.001 | 3,230 (29.1) |
| Yes | 7,717 (72.9) | 171 (32.1) |  | 7,888 (71.0) |
| *Medical care* |  |  |  |  |
| No | 1,942 (18.3) | 319 (60.0) | <0.001 | 2,261 (20.3) |
| Yes | 8,644 (81.7) | 213 (40.0) |  | 8,857 (79.7) |
| *Nursing and homecare* |  |  |  |  |
| No | 2,216 (20.9) | 323 (60.7) | <0.001 | 2,539 (22.8) |
| Yes | 8,370 (79.1) | 209 (39.3) |  | 8,579 (77.2) |
| *Cab drivers* |  |  |  |  |
| No | 3,534 (33.4) | 382 (71.8) | <0.001 | 3,916 (35.2) |
| Yes | 7,052 (66.6) | 150 (28.2) |  | 7,202 (64.8) |
| *Transportation* |  |  |  |  |
| No | 5,402 (51.0) | 444 (83.5) | <0.001 | 5,846 (52.6) |
| Yes | 5,184 (49.0) | 88 (16.5) |  | 5,272 (47.4) |
| *Retail* |  |  |  |  |
| No | 4,906 (46.3) | 415 (78.0) | <0.001 | 5,321 (47.9) |
| Yes | 5,680 (53.7) | 117 (22.0) |  | 5,797 (52.1) |
| *Lodging and leisure* |  |  |  |  |
| No | 4,014 (37.9) | 397 (74.6) | <0.001 | 4,411 (39.7) |
| Yes | 6,572 (62.1) | 135 (25.4) |  | 6,707 (60.3) |
| *Childcare* |  |  |  |  |
| No | 2,765 (26.1) | 370 (69.6) | <0.001 | 3,135 (28.2) |
| Yes | 7,821 (73.9) | 162 (30.5) |  | 7,983 (71.8) |
| *Hairdessing, beauty, and aesthetics* |  |  |  |  |
| No | 4,000 (37.8) | 392 (73.7) | <0.001 | 4,392 (39.5) |
| Yes | 6,586 (62.2) | 140 (26.3) |  | 6,726 (60.5) |
| *Government offices* |  |  |  |  |
| No | 4,638 (43.8) | 410 (77.1) | <0.001 | 5,048 (45.4) |
| Yes | 5,948 (56.2) | 122 (22.9) |  | 6,070 (54.6) |
| *Other income-earning jobs* |  |  |  |  |
| No | 7,244 (68.4) | 476 (89.5) | <0.001 | 7,720 (69.4) |
| Yes | 3,342 (31.6) | 56 (10.5) |  | 3,398 (30.6) |
| *Students* |  |  |  |  |
| No | 5,464 (51.6) | 446 (83.8) | <0.001 | 5,910 (53.2) |
| Yes | 5,122 (48.4) | 86 (16.2) |  | 5,208 (46.8) |
| *Homemakers* |  |  |  |  |
| No | 7,080 (66.9) | 482 (90.6) | <0.001 | 7,562 (68.0) |
| Yes | 3,506 (33.1) | 50 (9.4) |  | 3,556 (32.0) |
| *Other* |  |  |  |  |
| No | 10,368 (97.9) | 526 (98.9) | 0.136 | 10,894 (98.0) |
| Yes | 218 (2.1) | 6 (1.1) |  | 224 (2.0) |
| *None in particular* |  |  |  |  |
| No | 9,966 (94.1) | 391 (73.5) | <0.001 | 10,357 (93.2) |
| Yes | 620 (5.9) | 141 (26.5) |  | 761 (6.8) |
| **Do you support or oppose changing various activity restrictions depending on vaccination status (or whether or not one has proof of negative testing)?** |  |  |  |  |
| Support | 5,940 (56.1) | 135 (25.4) | <0.001 | 6,075 (54.6) |
| Oppose | 739 (7.0) | 156 (29.3) |  | 895 (8.1) |
| Neither support nor oppose | 3,907 (36.9) | 241 (45.3) |  | 4,148 (37.3) |
| **Which of the following would apply to you if the COVID-19 vaccination were made available to children under 12 years of age in the future?** |  |  |  |  |
| For (have children in the specified age range) | 716 (6.8) | 28 (5.3) | <0.001 | 744 (6.7) |
| For (do not have children in the specified age range) | 5,053 (47.7) | 97 (18.2) |  | 5,150 (46.3) |
| Against (have children in the specified age range) | 123 (1.2) | 36 (6.8) |  | 159 (1.4) |
| Against (do not have children in the specified age range) | 361 (3.4) | 78 (14.7) |  | 439 (4.0) |
| Neither for nor against (have children in the specified age range) | 412 (3.9) | 32 (6.0) |  | 444 (4.0) |
| Neither for nor against (do not have children in the specified age range) | 3,921 (37.0) | 261 (49.1) |  | 4,182 (37.6) |
| **Information sources** |  |  |  |  |
| **From what sources do you receive information about COVID-19? (Multiple Answer)** |  |  |  |  |
| *Physicians* |  |  |  |  |
| No | 8,634 (81.6) | 454 (85.3) | 0.028 | 9,088 (81.7) |
| Yes | 1,952 (18.4) | 78 (14.7) |  | 2,030 (18.3) |
| *Nurses* |  |  |  |  |
| No | 9,972 (94.2) | 508 (95.5) | 0.212 | 10,480 (94.3) |
| Yes | 614 (5.8) | 24 (4.5) |  | 638 (5.7) |
| *Pharmacists* |  |  |  |  |
| No | 10,258 (96.9) | 521 (97.9) | 0.177 | 10,779 (97.0) |
| Yes | 328 (3.1) | 11 (2.1) |  | 339 (3.1) |
| *Veterinarians* |  |  |  |  |
| No | 10,567 (99.8) | 529 (99.4) | 0.052 | 11,096 (99.8) |
| Yes | 19 (0.2) | 3 (0.6) |  | 22 (0.2) |
| *Dentists* |  |  |  |  |
| No | 10,472 (98.9) | 526 (98.9) | 0.912 | 10,998 (98.9) |
| Yes | 114 (1.1) | 6 (1.1) |  | 120 (1.1) |
| *Health fairs & events* |  |  |  |  |
| No | 10,502 (99.2) | 524 (98.5) | 0.078 | 11,026 (99.2) |
| Yes | 84 (0.8) | 8 (1.5) |  | 92 (0.8) |
| *Newspapers* |  |  |  |  |
| No | 5,886 (55.6) | 441 (82.9) | <0.001 | 6,327 (56.9) |
| Yes | 4,700 (44.4) | 91 (17.1) |  | 4,791 (43.1) |
| *Magazines* |  |  |  |  |
| No | 10,023 (94.7) | 505 (94.9) | 0.807 | 10,528 (94.7) |
| Yes | 563 (5.3) | 27 (5.1) |  | 590 (5.3) |
| *Books* |  |  |  |  |
| No | 10,278 (97.1) | 510 (95.9) | 0.104 | 10,788 (97.0) |
| Yes | 308 (2.9) | 22 (4.1) |  | 330 (3.0) |
| *Scientific literature* |  |  |  |  |
| No | 10,444 (98.7) | 523 (98.3) | 0.496 | 10,967 (98.6) |
| Yes | 142 (1.3) | 9 (1.7) |  | 151 (1.4) |
| *Television* |  |  |  |  |
| No | 1,336 (12.6) | 198 (37.2) | <0.001 | 1,534 (13.8) |
| Yes | 9,250 (87.4) | 334 (62.8) |  | 9,584 (86.2) |
| *Radio* |  |  |  |  |
| No | 9,041 (85.4) | 484 (91.0) | <0.001 | 9,525 (85.7) |
| Yes | 1,545 (14.6) | 48 (9.0) |  | 1,593 (14.3) |
| *Internet news sites* |  |  |  |  |
| No | 4,921 (46.5) | 307 (57.7) | <0.001 | 5,228 (47.0) |
| Yes | 5,665 (53.5) | 225 (42.3) |  | 5,890 (53.0) |
| *Search engines (Google, Yahoo, etc.)* |  |  |  |  |
| No | 8,264 (78.1) | 424 (79.7) | 0.374 | 8,688 (78.1) |
| Yes | 2,322 (21.9) | 108 (20.3) |  | 2,430 (21.9) |
| *LINE* |  |  |  |  |
| No | 9,897 (93.5) | 496 (93.2) | 0.814 | 10,393 (93.5) |
| Yes | 689 (6.5) | 36 (6.8) |  | 725 (6.5) |
| *Facebook* |  |  |  |  |
| No | 10,392 (98.2) | 521 (97.9) | 0.694 | 10,913 (98.2) |
| Yes | 194 (1.8) | 11 (2.1) |  | 205 (1.8) |
| *Twitter* |  |  |  |  |
| No | 9,985 (94.3) | 494 (92.9) | 0.156 | 10,479 (94.3) |
| Yes | 601 (5.7) | 38 (7.1) |  | 639 (5.8) |
| *Instagram* |  |  |  |  |
| No | 10,458 (98.8) | 516 (97.0) | <0.001 | 10,974 (98.7) |
| Yes | 128 (1.2) | 16 (3.0) |  | 144 (1.3) |
| *YouTube* |  |  |  |  |
| No | 10,124 (95.6) | 487 (91.5) | <0.001 | 10,611 (95.4) |
| Yes | 462 (4.4) | 45 (8.5) |  | 507 (4.6) |
| *TikTok* |  |  |  |  |
| No | 10,556 (99.7) | 525 (98.7) | <0.001 | 11,081 (99.7) |
| Yes | 30 (0.3) | 7 (1.3) |  | 37 (0.3) |
| *Medical information sites* |  |  |  |  |
| No | 10,277 (97.1) | 517 (97.2) | 0.894 | 10,794 (97.1) |
| Yes | 309 (2.9) | 15 (2.8) |  | 324 (2.9) |
| *Blogs or celebrity web pages* |  |  |  |  |
| No | 10,421 (98.4) | 520 (97.7) | 0.21 | 10,941 (98.4) |
| Yes | 165 (1.6) | 12 (2.3) |  | 177 (1.6) |
| *Local authorities such as prefectures and municipalities* |  |  |  |  |
| No | 6,289 (59.4) | 440 (82.7) | <0.001 | 6,729 (60.5) |
| Yes | 4,297 (40.6) | 92 (17.3) |  | 4,389 (39.5) |
| *Government* |  |  |  |  |
| No | 8,235 (77.8) | 473 (88.9) | <0.001 | 8,708 (78.3) |
| Yes | 2,351 (22.2) | 59 (11.1) |  | 2,410 (21.7) |
| *Medical task forces* |  |  |  |  |
| No | 9,437 (89.2) | 507 (95.3) | <0.001 | 9,944 (89.4) |
| Yes | 1,149 (10.9) | 25 (4.7) |  | 1,174 (10.6) |
| *Friends* |  |  |  |  |
| No | 8,864 (83.7) | 487 (91.5) | <0.001 | 9,351 (84.1) |
| Yes | 1,722 (16.3) | 45 (8.5) |  | 1,767 (15.9) |
| *Family members* |  |  |  |  |
| No | 8,260 (78.0) | 465 (87.4) | <0.001 | 8,725 (78.5) |
| Yes | 2,326 (22.0) | 67 (12.6) |  | 2,393 (21.5) |
| *Scientists and researchers* |  |  |  |  |
| No | 10,250 (96.8) | 515 (96.8) | 0.978 | 10,765 (96.8) |
| Yes | 336 (3.2) | 17 (3.2) |  | 353 (3.2) |
| *Pharmaceutical companies* |  |  |  |  |
| No | 10,445 (98.7) | 526 (98.9) | 0.688 | 10,971 (98.7) |
| Yes | 141 (1.3) | 6 (1.1) |  | 147 (1.3) |
| *Other companies* |  |  |  |  |
| No | 10,444 (98.7) | 476 (89.5) | <0.001 | 10,920 (98.2) |
| Yes | 142 (1.3) | 56 (10.5) |  | 198 (1.8) |
| **How much do you trust information about COVID-19 from the following sources? (4-point scale)** |  |  |  |  |
| Physicians | 2.8 (0.01) | 2.6 (0.03) | <0.001 | 2.8 (0.01) |
| Nurses | 2.7 (0.01) | 2.5 (0.03) | <0.001 | 2.6 (0.01) |
| Pharmacists | 2.5 (0.01) | 2.4 (0.03) | <0.001 | 2.5 (0.01) |
| Veterinarians | 2.1 (0.01) | 2.2 (0.03) | 0.001 | 2.1 (0.01) |
| Dentists | 2.2 (0.01) | 2.2 (0.03) | 0.994 | 2.2 (0.01) |
| Health fairs & events | 2.0 (0.01) | 2.1 (0.03) | 0.049 | 2.0 (0.01) |
| Newspapers | 2.4 (0.01) | 2.2 (0.03) | <0.001 | 2.4 (0.01) |
| Magazines | 2.0 (0.01) | 2.0 (0.03) | 0.165 | 2.0 (0.01) |
| Books | 2.1 (0.01) | 2.1 (0.03) | 0.453 | 2.1 (0.01) |
| Scientific literature | 2.4 (0.01) | 2.3 (0.03) | <0.001 | 2.4 (0.01) |
| Television | 2.3 (0.01) | 2.2 (0.03) | <0.001 | 2.3 (0.01) |
| Radio | 2.2 (0.01) | 2.1 (0.03) | <0.001 | 2.2 (0.01) |
| Internet news sites | 2.1 (0.01) | 2.1 (0.03) | 0.205 | 2.1 (0.01) |
| Search engines (Google, Yahoo, etc.) | 2.0 (0.01) | 2.1 (0.03) | 0.002 | 2.0 (0.01) |
| LINE | 1.7 (0.01) | 1.9 (0.03) | <0.001 | 1.7 (0.01) |
| Facebook | 1.5 (0.01) | 1.8 (0.03) | <0.001 | 1.5 (0.01) |
| Twitter | 1.5 (0.01) | 1.8 (0.03) | <0.001 | 1.5 (0.01) |
| Instagram | 1.5 (0.01) | 1.8 (0.03) | <0.001 | 1.5 (0.01) |
| YouTube | 1.5 (0.01) | 1.8 (0.03) | <0.001 | 1.6 (0.01) |
| TikTok | 1.4 (0.01) | 1.7 (0.03) | <0.001 | 1.4 (0.01) |
| Medical information sites | 2.2 (0.01) | 2.1 (0.03) | 0.006 | 2.2 (0.01) |
| Blogs or celebrity web pages | 1.6 (0.01) | 1.9 (0.03) | <0.001 | 1.6 (0.01) |
| Local authorities such as prefectures and municipalities | 2.5 (0.01) | 2.2 (0.03) | <0.001 | 2.5 (0.01) |
| Government | 2.4 (0.01) | 2.1 (0.03) | <0.001 | 2.4 (0.01) |
| Medical task forces | 2.5 (0.01) | 2.2 (0.04) | <0.001 | 2.5 (0.01) |
| Friends | 2.0 (0.01) | 2.1 (0.03) | 0.003 | 2.0 (0.01) |
| Family members | 2.3 (0.01) | 2.2 (0.03) | 0.423 | 2.3 (0.01) |
| Scientists and researchers | 2.4 (0.01) | 2.2 (0.03) | <0.001 | 2.4 (0.01) |
| Pharmaceutical companies | 2.3 (0.01) | 2.2 (0.03) | <0.001 | 2.3 (0.01) |
| Other companies | 1.9 (0.01) | 2.0 (0.03) | 0.006 | 1.9 (0.01) |
| **Do you believe the following COVID-19 information sources are sufficiently disseminated? (4-point scale)** |  |  |  |  |
| Physicians | 2.6 (0.01) | 2.6 (0.04) | 0.432 | 2.6 (0.01) |
| Nurses | 2.4 (0.01) | 2.5 (0.04) | 0.202 | 2.4 (0.01) |
| Pharmacists | 2.3 (0.01) | 2.4 (0.04) | 0.025 | 2.4 (0.01) |
| Veterinarians | 2.1 (0.01) | 2.3 (0.04) | <0.001 | 2.1 (0.01) |
| Dentists | 2.2 (0.01) | 2.4 (0.04) | <0.001 | 2.2 (0.01) |
| Health fairs & events | 2.2 (0.01) | 2.3 (0.04) | <0.001 | 2.2 (0.01) |
| Newspapers | 2.6 (0.01) | 2.4 (0.04) | <0.001 | 2.6 (0.01) |
| Magazines | 2.2 (0.01) | 2.3 (0.04) | 0.004 | 2.2 (0.01) |
| Books | 2.3 (0.01) | 2.4 (0.04) | 0.001 | 2.3 (0.01) |
| Scientific literature | 2.5 (0.01) | 2.4 (0.04) | 0.291 | 2.5 (0.01) |
| Television | 2.7 (0.01) | 2.5 (0.04) | <0.001 | 2.6 (0.01) |
| Radio | 2.5 (0.01) | 2.4 (0.04) | 0.004 | 2.5 (0.01) |
| Internet news sites | 2.4 (0.01) | 2.4 (0.04) | 0.561 | 2.4 (0.01) |
| Search engines (Google, Yahoo, etc.) | 2.3 (0.01) | 2.4 (0.04) | 0.176 | 2.4 (0.01) |
| LINE | 2.1 (0.01) | 2.2 (0.04) | <0.001 | 2.1 (0.01) |
| Facebook | 2.0 (0.01) | 2.2 (0.04) | <0.001 | 2.0 (0.01) |
| Twitter | 2.0 (0.01) | 2.2 (0.04) | <0.001 | 2.0 (0.01) |
| Instagram | 1.9 (0.01) | 2.2 (0.04) | <0.001 | 2.0 (0.01) |
| YouTube | 2.0 (0.01) | 2.2 (0.04) | <0.001 | 2.0 (0.01) |
| TikTok | 1.9 (0.01) | 2.1 (0.04) | <0.001 | 1.9 (0.01) |
| Medical information sites | 2.4 (0.01) | 2.4 (0.04) | 0.774 | 2.4 (0.01) |
| Blogs or celebrity web pages | 2.1 (0.01) | 2.2 (0.04) | <0.001 | 2.1 (0.01) |
| Local authorities such as prefectures and municipalities | 2.6 (0.01) | 2.4 (0.04) | <0.001 | 2.6 (0.01) |
| Government | 2.4 (0.01) | 2.3 (0.04) | 0.009 | 2.4 (0.01) |
| Medical task forces | 2.5 (0.01) | 2.4 (0.04) | <0.001 | 2.5 (0.01) |
| Friends | 2.2 (0.01) | 2.4 (0.04) | <0.001 | 2.3 (0.01) |
| Family members | 2.4 (0.01) | 2.5 (0.04) | 0.044 | 2.4 (0.01) |
| Scientists and researchers | 2.4 (0.01) | 2.4 (0.04) | 0.302 | 2.4 (0.01) |
| Pharmaceutical companies | 2.3 (0.01) | 2.3 (0.04) | 0.938 | 2.3 (0.01) |
| Other companies | 2.2 (0.01) | 2.3 (0.04) | <0.001 | 2.2 (0.01) |
| **To what extent did you consult information from the following sources in making your decision to vaccinate against COVID-19? (4-point scale)** |  |  |  |  |
| Physicians | 3.0 (0.01) | 2.7 (0.04) | <0.001 | 3.0 (0.01) |
| Nurses | 2.7 (0.01) | 2.5 (0.04) | <0.001 | 2.7 (0.01) |
| Pharmacists | 2.5 (0.01) | 2.4 (0.04) | 0.001 | 2.5 (0.01) |
| Veterinarians | 2.0 (0.01) | 2.1 (0.04) | <0.001 | 2.0 (0.01) |
| Dentists | 2.1 (0.01) | 2.2 (0.04) | 0.06 | 2.1 (0.01) |
| Health fairs & events | 2.1 (0.01) | 2.2 (0.04) | 0.004 | 2.1 (0.01) |
| Newspapers | 2.6 (0.01) | 2.3 (0.04) | <0.001 | 2.6 (0.01) |
| Magazines | 2.0 (0.01) | 2.1 (0.04) | 0.069 | 2.0 (0.01) |
| Books | 2.1 (0.01) | 2.2 (0.04) | 0.015 | 2.1 (0.01) |
| Scientific literature | 2.3 (0.01) | 2.3 (0.04) | 0.322 | 2.3 (0.01) |
| Television | 2.8 (0.01) | 2.5 (0.04) | <0.001 | 2.8 (0.01) |
| Radio | 2.3 (0.01) | 2.2 (0.04) | 0.015 | 2.3 (0.01) |
| Internet news sites | 2.4 (0.01) | 2.4 (0.04) | 0.507 | 2.4 (0.01) |
| Search engines (Google, Yahoo, etc.) | 2.3 (0.01) | 2.3 (0.04) | 0.171 | 2.3 (0.01) |
| LINE | 1.8 (0.01) | 2.0 (0.04) | <0.001 | 1.8 (0.01) |
| Facebook | 1.7 (0.01) | 1.9 (0.04) | <0.001 | 1.7 (0.01) |
| Twitter | 1.7 (0.01) | 2.0 (0.04) | <0.001 | 1.7 (0.01) |
| Instagram | 1.6 (0.01) | 1.9 (0.04) | <0.001 | 1.6 (0.01) |
| YouTube | 1.7 (0.01) | 2.0 (0.04) | <0.001 | 1.7 (0.01) |
| TikTok | 1.6 (0.01) | 1.9 (0.04) | <0.001 | 1.6 (0.01) |
| Medical information sites | 2.2 (0.01) | 2.1 (0.04) | 0.379 | 2.2 (0.01) |
| Blogs or celebrity web pages | 1.7 (0.01) | 2.0 (0.04) | <0.001 | 1.8 (0.01) |
| Local authorities such as prefectures and municipalities | 2.8 (0.01) | 2.4 (0.04) | <0.001 | 2.8 (0.01) |
| Government | 2.7 (0.01) | 2.3 (0.04) | <0.001 | 2.7 (0.01) |
| Medical task forces | 2.7 (0.01) | 2.3 (0.04) | <0.001 | 2.7 (0.01) |
| Friends | 2.2 (0.01) | 2.3 (0.04) | 0.34 | 2.2 (0.01) |
| Family members | 2.5 (0.01) | 2.4 (0.04) | 0.138 | 2.4 (0.01) |
| Scientists and researchers | 2.4 (0.01) | 2.3 (0.04) | 0.001 | 2.4 (0.01) |
| Pharmaceutical companies | 2.3 (0.01) | 2.2 (0.04) | 0.067 | 2.3 (0.01) |
| Other companies | 2.0 (0.01) | 2.1 (0.04) | 0.001 | 2.0 (0.01) |

**STable 4. Odds ratios (95% confidence intervals) for remaining unvaccinated among those with initial vaccine willingness.**

|  | **Odds Ratio (95% CI)** | **p** |
| --- | --- | --- |
| **Socioeconomic** |  |  |
| **Marital Status** |  |  |
| Unmarried | Ref. |  |
| Married | 0.64 (0.51-0.80) | <0.001 |
|  |  |  |
| **Health** |  |  |
| **Do you receive influenza vaccines?** |  |  |
| Every year | Ref. |  |
| Every few years | 1.35 (1.01-1.81) | 0.041 |
| Rarely or never | 2.08 (1.63-2.66) | <0.001 |
|  |  |  |
| **How confident are you when filling out medical forms by yourself? (health literacy)** |  |  |
| Not at all | Ref. |  |
| A little bit | 0.76 (0.34 to 1.69) | 0.496 |
| Somewhat | 0.81 (0.40 to 1.64) | 0.556 |
| Fairly | 0.53 (0.26 to 1.06) | 0.073 |
| Extremely | 0.46 (0.22 to 0.93) | 0.032 |
|  |  |  |
| **What is your best guess as to whether you will get COVID-19 within the next 6 months?** |  |  |
| I don't think I will get COVID-19 | Ref. |  |
| I think I will get a mild case of COVID-19 | 0.79 (0.62-1.00) | 0.054 |
| I think I will get seriously ill from COVID-19 | 1.32 (0.95-1.83) | 0.104 |
| I have already had COVID-19 | 2.98 (1.69-5.24) | <0.001 |
|  |  |  |
| **Have you ever received a COVID-19 test?** |  |  |
| Yes | Ref. |  |
| No | 1.38 (1.08-1.78) | 0.011 |
|  |  |  |
| **Do you engage in preventive measures against COVID-19 (e.g., masking, minimizing outings, etc.)?** |  |  |
| Yes | Ref. |  |
| No | 2.31 (1.55-3.44) | <0.001 |
|  |  |  |
| **Psychology and attitudes** |  |  |
| **How do you feel are the benefits of the COVID-19 vaccine? (perceived benefits of the COVID-19 vaccine)** |  |  |
| Very small | Ref. |  |
| Small | 0.45 (0.24 to 0.85) | 0.014 |
| Medium | 0.35 (0.19 to 0.64) | 0.001 |
| Large | 0.17 (0.09 to 0.32) | <0.001 |
| Very large | 0.24 (0.12 to 0.47) | <0.001 |
|  |  |  |
| **How do you think the disadvantages of the COVID-19 vaccine are? (perceived risks of the COVID-19 vaccine)** |  |  |
| Very small | Ref. |  |
| Small | 1.50 (0.83 to 2.73) | 0.179 |
| Medium | 2.65 (1.46 to 4.79) | 0.001 |
| Large | 3.98 (2.17 to 7.30) | <0.001 |
| Very large | 4.45 (2.23 to 8.90) | <0.001 |
|  |  |  |
| **If others have been vaccinated against COVID-19, I believe I should be vaccinated as well** |  |  |
| Strongly disagree | Ref. |  |
| Disagree | 0.57 (0.34 to 0.96) | 0.034 |
| Neither agree nor disagree | 0.29 (0.18 to 0.47) | <0.001 |
| Agree | 0.13 (0.08 to 0.22) | <0.001 |
| Strongly agree | 0.10 (0.06 to 0.19) | <0.001 |
|  |  |  |
| **If you have already been vaccinated, how many people around you were vaccinated at the time you received the first dose, and if not, how many people around you are currently vaccinated?** |  |  |
| About 0% | Ref. |  |
| About 25% | 0.42 (0.27-0.66) | <0.001 |
| About 50% | 0.58 (0.38-0.87) | 0.008 |
| About 75% | 0.44 (0.30-0.64) | <0.001 |
| About 100% | 0.26 (0.17-0.40) | <0.001 |
|  |  |  |
| **For which professions do you believe vaccination should be prioritized? (multiple answer)^*^** |  |  |
| Office workers | 0.71 (0.56-0.92) | 0.009 |
| Medical care | 0.54 (0.43-0.68) | <0.001 |
|  |  |  |
| **Do you support or oppose changing various activity restrictions depending on vaccination status (or whether or not one has proof of negative testing)?** |  |  |
| Support | Ref. |  |
| Neither support nor oppose | 1.34 (1.03 to 1.73) | 0.028 |
| Oppose | 2.63 (1.92 to 3.61) | <0.001 |
|  |  |  |
| **Which of the following would apply to you if the COVID-19 vaccination were made available to children under 12 years of age in the future?** |  |  |
| For (have children in the specified age range) | Ref. |  |
| For (do not have children in the specified age range) | 0.89 (0.55 to 1.47) | 0.660 |
| Neither for nor against (have children in the specified age range) | 1.71 (0.93 to 3.13) | 0.084 |
| Neither for nor against (do not have children in the specified age range) | 1.34 (0.85 to 2.22) | 0.188 |
| Against (have children in the specified age range) | 2.98 (1.59 to 5.61) | 0.001 |
| Against (do not have children in the specified age range) | 1.41 (0.79 to 2.50) | 0.242 |
|  |  |  |
| **Information sources** |  |  |
| **From what sources do you receive information about COVID-19? (multiple answer)^*^** |  |  |
| *Pharmacists* | 0.37 (0.17-0.81) | 0.012 |
| *Newspapers* | 0.62 (0.47-0.82) | 0.001 |
| *Magazines* | 2.43 (1.47-4.01) | 0.001 |
| *Twitter* | 0.57 (0.37-0.89) | 0.013 |
| *YouTube* | 1.72 (1.11-2.65) | 0.015 |
| *Local authorities such as prefectures and municipalities* | 0.61 (0.47-0.81) | 0.001 |
| *Other companies* | 3.19 (2.03-5.00) | <0.001 |
|  |  |  |
| **How much do you trust information about COVID-19 from the following sources? (4-point scale)** |  |  |
| Scientific literature | 0.70 (0.58-0.84) | <0.001 |
| YouTube | 1.37 (1.15-1.62) | <0.001 |
| Pharmaceutical companies | 1.38 (1.14-1.68) | 0.001 |
|  |  |  |
| **Do you believe the following COVID-19 information sources are sufficiently disseminated? (4-point scale)** |  |  |
| Dentists | 1.21 (1.06-1.38) | 0.005 |

^*^ The reference categories for multiple answer questions were those who did not select any given answer choice.

**References**

1. Mutambudzi M, Niedwiedz C, Macdonald EB, et al. Occupation and risk of severe COVID-19: prospective cohort study of 120 075 UK Biobank participants. *Occup Environ Med*. Dec 9 2020;doi:10.1136/oemed-2020-106731

2. CISA. Identifying Critical Infrastructure During COVID-19. Cybersecurity & Infrastructure Security Agency. Accessed May 26, 2022, <https://www.cisa.gov/identifying-critical-infrastructure-during-covid-19>
